# Optimizing image watermarking integrity and visual quality via DTPSO and hybrid transform methods

**DOI:** 10.1038/s41598-026-49253-0

**Published:** 2026-04-21

**Authors:** Doaa Sami Khafaga, El-Sayed M. El-kenawy, Nima Khodadadi, Marwa M. Eid, Seyedali Mirjalili

**Affiliations:** 1 Department of Computer Sciences, College of Computer and Information Sciences, Princess Nourah bint Abdulrahman University, P.O. Box 84428, Riyadh, 11671 Saudi Arabia; 2 Department of Communications and Electronics, Delta Higher Institute of Engineering and Technology, Mansoura 35111, Egypt; 3 Jadara Research Center, Jadara University, Irbid, 21110 Jordan; 4 Department of Civil and Environmental Engineering, Pacific Earthquake Engineering Research (PEER) Center, University of California, Berkeley, CA USA; 5 Faculty of Artificial Intelligence, Delta University for Science and Technology, Mansoura, 11152 Egypt; 6 Centre for Artificial Intelligence Research and Optimisation, Torrens University Australia, Fortitude Valley, Brisbane, QLD 4006 Australia

**Keywords:** Discrete cosine transform, Dipper throated optimization, Particle swarm optimization, Noise reduction, Discrete wavelet transform, Engineering, Mathematics and computing

## Abstract

Image watermarking is an important extension of intellectual property protection that facilitates the identification and authentication of multimedia content. This paper aims to improve and optimize image watermarking techniques to ensure effective image protection regardless of image size or format. The proposed framework integrates discrete wavelet transform (DWT) and discrete cosine transform (DCT) to enhance watermark embedding performance and preserve data integrity. In addition, the study addresses a common challenge in watermarking systems, namely the increase in perceptible noise that may degrade visual image quality after watermark embedding. To overcome this issue, advanced noise-reduction and feature-processing strategies are incorporated, including AlexNet-based feature extraction, principal component analysis (PCA), independent component analysis (ICA), blind source separation (BSS), and optimization-assisted BSS. Extensive experiments are conducted to evaluate the effectiveness of the proposed method in terms of imperceptibility, robustness, extraction reliability, and computational efficiency. The proposed Dipper-Throated Particle Swarm Optimization (DTPSO) algorithm combined with DWT-DCT achieves a peak signal-to-noise ratio (PSNR) of 65.78 dB, a normalized cross-correlation (NCC) of 0.9189, an accuracy of 0.9766, and a bit error rate (BER) of 0.0234 on color images, demonstrating strong watermark imperceptibility and reliable extraction performance under different attack conditions.In addition, the proposed DWT + DCT+DTPSO model exhibits superior computational efficiency, achieving the lowest execution time of 46.5 s and the minimum memory consumption of 768 MB among the compared methods. These results confirm that the proposed methodology provides an effective and efficient image watermarking solution that enhances the protection, robustness, and integrity of digital multimedia content.

## Introduction

Digital watermarking is vital to security watermarking across various intellectual property rights matters involving multimedia formats. Implementing this technology means embedding small but powerful marks into digital content to protect copyright, verify authenticity, and confirm data accuracy^1^. Finding the right solution that perfectly combines the two terms, imperceptibility and robustness, is one of the most challenging tasks in watermarking^2^. What is difficult is the development of robust algorithms capable of standing a significant number of attacks and still not noticed by human eyes. The attacks may vary not only in their nature but also in intensity and the target area. They may comprise an array of operations, including reduction, noise addition, filtering, cropping, and geometric distortions^3^. Every kind of destruction affects the watermarking approach because of the complexity of the tasks required and the system’s effective evolutionary strategy. The noise influence may become a problem that should be carefully considered after image post-watermarking. Sound integration is sometimes accompanied by increased noise, which harms the resulting image and blurs the watermark^4^. Compression algorithms, transmission errors, and limitations of human visual perception further attenuate this effect^5^.

The researchers have devised many ways of resolving these problems, including various techniques and implementation methods for digital watermarks. Some of the most prominent methods include transform-domain methods, in which mathematical transformation techniques such as the discrete Fourier transform (DFT), discrete wavelet transform (DWT), and discrete cosine transform (DCT) are applied^6,7^. Based on either the frequency or the spatial domain, these transformations allow us to embed the data safely as a watermark while preserving the perception of the image. Another approach that has become usual for watermarking developers is matrix decomposition, especially the singular value decomposition (SVD). This practice has proved helpful for ensuring the stability of the watermarking process against attacks^8^. A mirror process of forming images into a single value and a vector coefficient using SVD-based methods makes them powerful and robust for various operations in image processing, copyright protection, and content authentication applications^9^.

Furthermore, methods of metaheuristic optimization have been developed as potential weapons to enhance watermark protection and improve overall performance and resilience. Algorithms such as particle swarm optimization (PSO), genetic algorithms (GA), and simulated annealing (SA) enable the harmonization of watermark embedding parameters so that the watermark is barely noticeable yet robust to attacks^10,11^. However, the increased noise in the watermarking process led to the development of novel noise-reduction techniques. Such techniques can achieve this purpose by applying advanced signal processing and machine learning methods, optimizing noise-reduction methods, and maintaining the image’s high quality and the watermark’s invisibility^12^.

Feature extraction can be performed using deep learning models such as AlexNet. This is one of the most preferred train designs^13^. By taking features like edges and contours of images at a high level, deep learning models help to convert images into numbers, which is beneficial when it comes to noise reduction techniques such as Principal Component Analysis (PCA), Independent Component Analysis (ICA), and Blind Source Separation (BSS)^14^. While PCA, ICA, and BSS are very effective for removing noise from watermarked images, their main disadvantage is increased computational overhead, which can be mitigated with efficient algorithms. PCA is beneficial because it identifies the most significant components of the dataset’s variation while removing excess noise and mildly altering image details^15^. ICA also allows separating mixed signals by decomposing them into statistically independent components, removing their interference from the noise^16^. Unlike conventional techniques, BSS filters mix signals from various sources without explicit knowledge of the mixing process, making them effective for noise reduction^17^.

This work addresses the challenge of noise in watermarked images by applying cutting-edge nonlinear noise reduction approaches and optimization tools. We consider a broad-based algorithm that combines transform-domain methodologies, matrix decomposition techniques, metal-guy optimization approaches, and advanced noise-reduction methods.

**The contributions are as follows**:

- Introduces advanced watermarking techniques using DWT and DCT optimized with Dipper-Throated Particle Swarm Optimization (DTPSO), achieving superior performance with improved image quality and security.
- Explores noise reduction techniques using AlexNet, PCA, ICA, and BSS that preserve watermark structure while maintaining low bit error rates under various attack conditions.
- Optimizes BSS using DTPSO-BSS, demonstrating significant improvements in noise suppression and robust watermark extraction across multiple attack scenarios.
- Conducts comprehensive statistical analysis using ANOVA and Wilcoxon signed rank tests, with DTPSO-BSS consistently outperforming traditional methods, including DFT + SVD, DWT + DCT, and other optimization-based techniques.

The remainder of this paper is organized as follows: Section 2 presents related work in digital watermarking, describing existing techniques. Section  3 focuses on the theoretical background and the methods used in our study. Section  4 focuses on the intended methodology to help reduce and improve the noise in digital watermarking. Section  5 presents the empirical results and the evaluation method. Section  6, which concludes this paper, is an overview of the key findings and future research recommendations.

## Related works

As a multimedia security technique, digital watermarking has proven to be of great importance, particularly for copyright protection, content authentication, and secure data transmission. Since digital media has become easier to duplicate and manipulate, robust, imperceptible watermarking schemes have become a central research focus. Recently, transform-domain techniques and a nature-inspired optimization algorithm were combined to improve the invisibility and robustness of watermarking systems significantly.

An optimal blind color image watermarking approach based on TVT, LWT, and Schur decomposition optimized via the Adaptive Chaotic Grasshopper Optimization Algorithm has been introduced by Prabha and Shatheesh^18^. They employ Schur decomposition on the middle-frequency components of the wavelet domain (HL and LH) to embed the watermark and achieve some increase in robustness. Chaotic maps are used for watermark encryption to further enhance security, resist various geometric attacks, and achieve a PSNR of 54.2987 dB, a very high value indicating excellent perceptual quality.

Optimal embedding factor selection remains a challenging issue in video watermarking; as reported in^19^, this problem was addressed using a modified gravitational search algorithm (MGSA)– based watermarking scheme that adaptively determines multiple optimal embedding factors. The proposed approach embeds the watermark in high-entropy blocks using RDWT and Schur decomposition and, through extensive experimental evaluation, was shown to achieve a superior balance between imperceptibility and robustness compared with existing methods.

The Grasshopper optimization algorithm was developed by Cheemalapati et al.^20^, and a combination of the Grasshopper optimization algorithm and a genetic algorithm was proposed to achieve better performance than both algorithms, known as the hybrid grasshopper optimization and genetic algorithm (HGOAGA). In contrast to single algorithms, hybridization helps overcome the exploration-exploitation trade-off, achieving greater proficiency in optimizing watermark placement in the DWT and SVD domains. Because of the synergy between GOA’s global search and GA’s mutation and crossover mechanisms, their method is shown to be more robust against common image processing attacks.

Yıldız et al.^21^ proposed a digital image watermarking method based on a hybrid transform-domain approach combining DWT, DCT, and SVD. Bacterial Foraging Optimization (BFO) is used in the framework, along with Particle Swarm Optimization (PSO), to fine-tune the chemotactic parameters in BFO and the watermark embedding process. We present their results demonstrating their superiority in resisting noise-based attacks, such as Gaussian, salt-and-pepper, and speckle noise, compared to traditional optimization techniques.

In^22^, Kumari et al. presented a watermarking scheme using the DWT— SVD and the Enhanced Tunicate Swarm Optimization Algorithm (TSA). The Sine Cosine Algorithm (SCA) principles are incorporated into this enhanced TSA to improve optimizer convergence behavior. In addition, the scheme relies on Arnold scrambling and Tent Map chaotic encryption to provide a dual security layer, underscoring the importance of encryption in ensuring the integrity of the watermark, alongside optimization. Their approach was experimentally robust across several image datasets and attack scenarios accordingly.

Optimal selection of the embedding factor for preserving the integrity and authenticity of electronic patient records was addressed by^23^ using a hybrid IWT–SVD watermarking scheme optimized with NSGA-II. The method employs chaotic encryption and multi-objective optimization to balance imperceptibility and robustness, achieving improved PSNR and NC performance under various attack scenarios while remaining false-positive-free.

Cavagnino et al.^24^ have presented a method for high-capacity reversible data hiding in radiographic images by optimizing bit allocation using combinatorial optimization techniques in the context of reversible watermarking. They address the specific challenges of medical images, where reversibility and distortion minimization are critical. Optimization algorithms are applied to optimize designs, maximizing payload capacity while ensuring watermark image recovery, highlighting the growing importance of optimization strategies in watermarking systems.

Awasthi and Srivastava^25^ extended watermarking research to the medical domain using Hessenberg decomposition, along with LWT and SVD. The optimization process for the PSO and JAYA algorithms determines optimal scaling factors for watermark embedding. Advanced Encryption Standard (AES) was also applied for watermark authentication, thereby meeting the requirements of hybrid systems that seek to incorporate optimization, transform-domain techniques, and encryption for higher security.

Similarly, Koolwal and Sharma^26^ propose an optimization-driven watermarking approach within a hybrid DWT–DCT–SVD framework. Maximum fitness function was obtained by applying PSO and JAYA algorithms in such a way as to improve robustness and imperceptibility. Considering this, we compare our results with those of other optimization algorithms and demonstrate the flexibility and effectiveness of combining different metaheuristic strategies to solve watermarking problems.

Lastly, Agarwal et al.^27^ proposed a grayscale image watermarking framework for optimizing multiple scaling factors in the hybrid transform domain using the Harmony Search Algorithm (HSA). The improvisation process of musicians by HSA provides an innovative search strategy to balance watermark invisibility and robustness. They show that their results are highly resilient to the most common image processing attacks, except for cropping, which remains to be improved.

Table 1 provides a comprehensive overview of recent literature on digital image watermarking, highlighting the prevalence of hybrid transform-domain techniques, such as DWT, DCT, SVD, and Schur decomposition, combined with nature-inspired metaheuristic optimization algorithms. The reviewed studies demonstrate that hybrid approaches consistently outperform single-method techniques, achieving high PSNR values exceeding 54 dB and enhanced robustness against various attacks while also providing additional security through chaotic encryption methods.

**Table 1:** Summary of literature review.

Reference	Methods/Techniques	Key Findings/Performance
Ref^18^.	TVT, LWT, Schur decomposition, Adaptive Chaotic Grasshopper Optimization Algorithm	PSNR of 54.2987 dB; robust against geometric attacks with chaotic encryption
Ref^19^.	Modified Gravitational Search Algorithm (MGSA), RDWT, Schur decomposition	Adaptive multiple embedding factors for video watermarking; superior imperceptibility-robustness balance
Ref^20^.	Hybrid Grasshopper Optimization and Genetic Algorithm (HGOAGA), DWT, SVD	Enhanced robustness against common image processing attacks through hybrid optimization
Ref^21^.	DWT, DCT, SVD, Bacterial Foraging Optimization (BFO), PSO	Superior resistance to noise-based attacks, including Gaussian, salt & pepper, and speckle noise
Ref^22^.	DWT, SVD, Enhanced Tunicate Swarm Optimization (TSA), SCA, Arnold scrambling, Tent Map encryption	Robust across multiple image datasets and attack scenarios with a dual security layer
Ref^23^.	IWT, SVD, NSGA-II, chaotic encryption	Optimal for medical records; improved PSNR and NC under various attacks; false-positive-free
Ref^24^.	Reversible data hiding, combinatorial optimization	High-capacity reversible watermarking for medical images with minimized distortion
Ref^25^.	Hessenberg decomposition, LWT, SVD, PSO, JAYA, AES encryption	Medical domain watermarking with optimal scaling factors and advanced encryption
Ref^26^.	DWT, DCT, SVD, PSO, JAYA	Enhanced robustness and imperceptibility through multi-metaheuristic optimization
Ref^27^.	Harmony Search Algorithm (HSA), hybrid transform domain	Strong resilience to common attacks except cropping; balanced invisibility and robustness

## Materials and methods

This section describes the fundamental approaches that constitute the proposed image watermarking framework. The methodology combines transform-domain techniques—specifically, the discrete wavelet transform (DWT) and the discrete cosine transform (DCT)—with nature-inspired metaheuristic algorithms, namely dipper-throated optimization (DTO) and particle swarm optimization (PSO). These methods work synergistically to optimize watermark imperceptibility, robustness, and noise suppression. Theoretical backgrounds, mathematical formulations, and algorithmic details for each technique are presented.

### Transform domain methods

#### Discrete wavelet transform (DWT)

In terms of watermarking, DWT is a standard method after the FWT [28]. DWT is an effective tool for analyzing signals by decomposing them into several frequency sub-bands, which can then represent the signal at different granularities. By applying the same concept with greater precision, the watermark can now be embedded in the least perceptible frequency sub-bands. Compared to DCT in watermarking, DWT excels in scenarios involving signal compression or noise addition. The DCF-to-DWT-related watermarks are more complicated to implement and require more computational resources than DCT. Nevertheless, the DWT-watermarking techniques have shown successful applications in several domains of multimedia, such as multimedia authentication, copyright protection, and content-based retrieval.

The wavelet transform is a space-time analytic tool adaptable to any signal’s time-frequency properties. The time-frequency scaling and translation representation of the signal is realized by the wavelet transform, which divides the spectrum using telescoping translations at both ends. When we assume that ψ(t) is a fundamental or mother wavelet, the generalized wavelet transform is expressed in Eq. (1), where the scaling parameter a and translation parameter b allow flexible signal analysis through customizable mother waves.

1 $$\:\phi\:\left(t\right)=\frac{1}{\sqrt{\left|a\right|}}\psi\:\left(\frac{(t-b)}{a}\right),a\ne\:0$$

Wavelet transformation in the continuous domain takes quite a long time. It provides a large amount of data by calculating wavelet coefficients across all available scales. The discrete wavelet coefficient function f(k) is presented in Eq. (2):

2 $$\:DWT(w,h)={2}^{\frac{-w}{h})}\sum\:_{k=-\mathrm{i}\mathrm{n}\mathrm{f}}^{\mathrm{i}\mathrm{n}\mathrm{f}} f\left(k\right){\phi\:}^{\mathrm{*}}({2}^{-w}k-h)$$

where ϕ* is the complex conjugate of ψ(t), which represents the coordinate of the discretized point in the discrete space denoted by w and h. Using powers of two for upscaling and parsing does not impair the precision but facilitates the analysis. According to researchers, wavelet analysis is done through a series of wavelet bases. It emulates the process of collecting and reconstructing images after decomposition (Morlet, Daubechies, Haar). The Haar wavelet function, defined in Eq. (3), is used as the multi-resolution decomposed basis function.

3 $$\:\psi\:\left(t\right)=\left\{\begin{array}{ll}1&\:0\le\:t\le\:\frac{1}{2}\\\:-1&\:\frac{1}{2}\le\:t<1\\\:0&\:other\\\:&\:\end{array}\right.$$

**Fig. 1:**
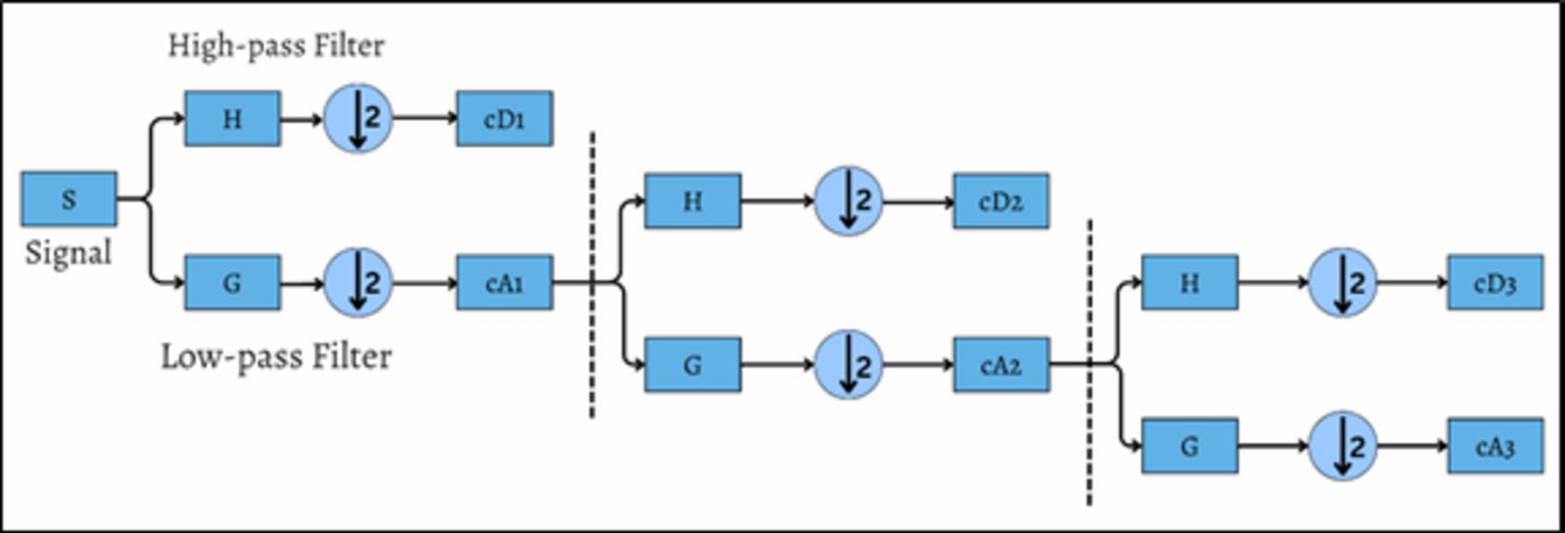
Three-level decomposition of a one-dimensional signal using DWT.

Figure 1 is presented visually as a DWT (Discrete Wavelet Transform), a data-adaptive, multi-resolution analysis used as a wavelet subspace basis in the signal processing system. We use a dual-band (DWT) filter to separate the high- and low-frequency bands present in the original signal, represented by the filters G and H.

**Fig. 2:**
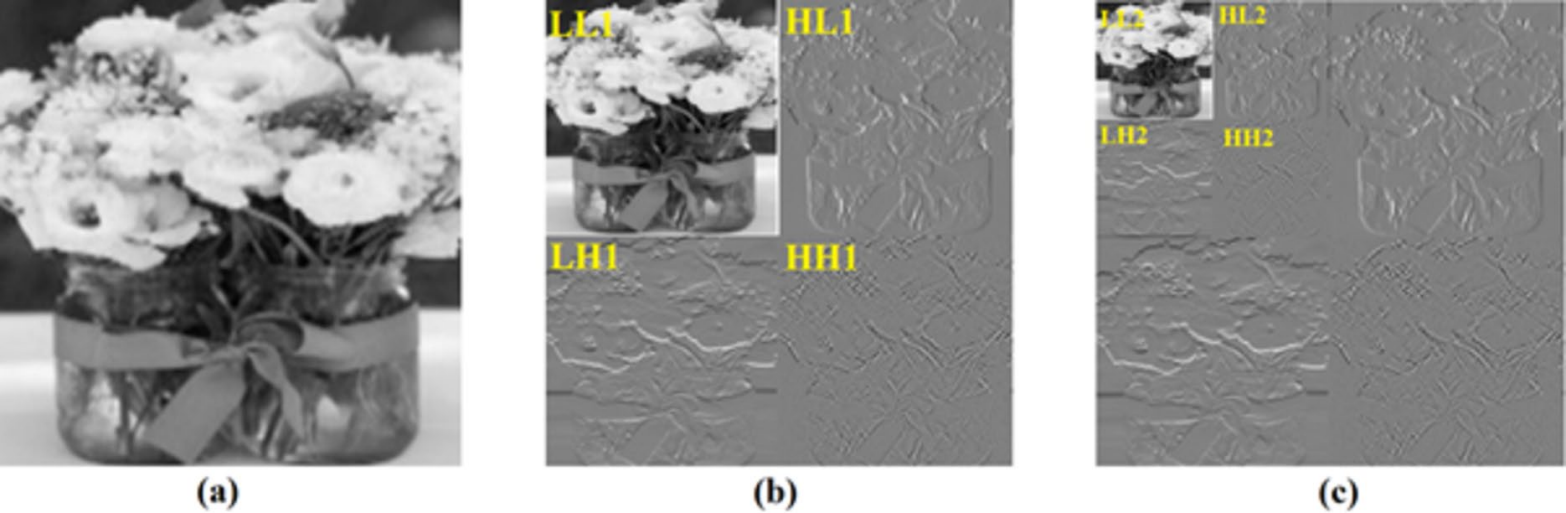
Two-Level DWT decomposition of two-dimensional image.

Figure 2 provides additional context for the DWT’s use in signal processing algorithms, as outlined in the application section. In particular, it shows the subbands and uses the G and H symbols for the high-pass and low-pass filtering. The DWT is illustrated by the representation of spatiotemporal data at multiple resolutions, which highlights its optimal adaptive capacity.

#### Discrete cosine transform (DCT)

Digital watermarking is one of the most commonly used techniques for inserting a watermark that operates in the frequency domain, and it is often referred to as DCT [29]. Though the DCT can be employed to compress a signal into a set of straightforwardly adjustable frequency coefficients, changes to these coefficients incur only a negligible loss of information, making it particularly suitable for watermarking. The imperceptibility of the watermark is determined by distorting the quantized coefficients imperceptibly for a regular listener or eye, and a watermark detector identifies them. The watermark, as such, could be utilized in various ways, namely, copyright protection, authentication, and identification of pirated copies. In light of their dependability, efficiency, and simplicity, DCT-based watermarking systems have seen widespread adoption in recent years. The DCT is connected with the Fourier transform. However, its output of real-valued coefficients is more beneficial than Fourier coefficients for applying it to data compression and encoding.

The discrete cosine transform decomposes a signal into the frequency domain. Sinusoids of different amplitudes and frequencies define how wave patterns move about the image. When DCT is used to convert an original image, denoted by x, to a new image, also denoted by y, Eq. (4) is applied to determine the DCT coefficients. In the formula, x is denoted by a stand-alone variable for an N by M pixel input image. x(m, n) is a notation used to represent a pixel intensity for the picture in the row m and column n of the DCT matrix, and y(u, v) is a notation for a DCT coefficient in the row u and column v of the DCT matrix.

4 $$\:y(u,v)=\sqrt{\frac{2}{M}}\sqrt{\frac{2}{N}}{A}_{u}{A}_{v}\sum_{u=0}^{M-1} \sum_{v=0}^{N-1}x(m,n)cos\frac{(2m+1)\pi\:}{2M}cos\frac{(2n+1)v\pi\:}{2N}$$

Where the normalization factors $\:{A}_{u}$and $\:{A}_{v}\:$are defined in Eqs. (5) and (6):

5 $$\:{\mathrm{W}\mathrm{h}\mathrm{e}\mathrm{r}\mathrm{e}:A}_{u}=\left\{\begin{array}{ll}\frac{1}{\sqrt{2}}&\:u=0\\\:1&\:u=\mathrm{1,2},3,...,N-1\\\:&\:\end{array}\right.$$

6 $$\:\mathrm{W}\mathrm{h}\mathrm{e}\mathrm{r}\mathrm{e}:\:{A}_{v}=\left\{\begin{array}{ll}\frac{1}{\sqrt{2}}&\:v=0\\\:1&\:v=\mathrm{1,2},3,...,N-1\\\:&\:\end{array}\right.$$

Inverse DCT is used to reconstruct the image in accordance with Eq. (7):

7 $$\:x(m,n)=\sqrt{\frac{2}{M}}\sqrt{\frac{2}{N}}\sum_{u=0}^{M-1}\sum_{v=0}^{N-1}{A}_{u}{A}_{v}y(u,v)cos\frac{(2m+1)\pi\:}{2M}cos\frac{(2n+1)v\pi\:}{2N}$$

The standard operation process consists of slicing a picture into non-overlapping parcels and then transforming each parcel by applying the DCT independently. This technique has three frequency sub-bands, generating low, intermediate, and high frequencies, just as the corresponding sub-bands do. Two fundamental principles can summarize the background of DCT-based watermarking. Less crucial visual information about an image appears in the high-frequency subband, which also contains more signal energy. Noise, especially high-frequency noise, is more prone to manual errors. The secret watermark is securely preserved by fine-tuning the coefficients of the mid-frequency sub-band, which relates to these problems the most. This approach sheds light on image ethnicity losses that can occur during compression, while preserving watermark identification and image readability. One of them is that the shift occurs from the temporal-frequency domain to the spatial-frequency domain, hence increasing the block length of the watermarking. Not only does the versatility and adaptability.

The watermark needs to be embedded in an image area (such as a mid-frequency sub-band) that remains resilient to compression and noise interference, ensuring high-quality image preservation. The placement of the watermark aligns with human visual perception, allowing it to remain hidden without degrading image quality. The technique ensures both visibility and protection by targeting less sensitive areas of the visual field to maintain image attractiveness. The application of block-based DFT to choose appropriate frequency bands strengthens watermark security while minimizing visibility and protecting digital content from unauthorized changes or theft.

### Optimization algorithms

#### Dipper throated optimization (DTO)

This approach involves utilizing a group of potential solutions, referred to as dippers in the study. This is done by the dipper-throated optimization (DTO) algorithm [30]. In general, “dippers” are the decision variables that constitute a set of alternatives to choose the best course of action based on optimal strategies. Different dibbers’ actions are regulated by the respective sets of adaptable rules, which are flexible and can change their search approaches depending on the current environment. During the algorithm’s execution or during iterations, the agents explore numerous areas of the search space until they find the best fit.

The DTO algorithm optimizes performance through three integrated adaptive mechanisms: local search explores neighborhoods within a step-size-controlled radius; global search enables information sharing among all dippers through adjustable flight ranges; and memory search utilizes previously discovered optimal solutions with a configurable memory size. The combined movement of each dipper emerges from these three strategies, with adaptive parameters continuously updated during execution to balance exploration and exploitation.

The algorithm operates on a population of m dippers in d-dimensional search space, where the position matrix X and velocity matrix Y represent the swarm’s state, as shown in Eqs. (8) and (9), respectively. In the jth dimension, where i ∈ {1,2,3,…,m} and j ∈ {1,2,3,…,d}, the ith bird is designated by $\:{X}_{i,j}\:$and its velocity is represented by by $\:{Y}_{i,j}\:.$ The fitness array h = {h₁, h₂, h₃,…, hₙ} calculates each bird’s objective function value, as defined in Eq. (10).

8 $$\:X=\left[\begin{array}{lllll}{X}_{\mathrm{1,1}}&\:{X}_{\mathrm{1,2}}&\:{X}_{\mathrm{1,3}}&\:...&\:{X}_{1,d}\\\:{X}_{\mathrm{2,1}}&\:{X}_{\mathrm{2,2}}&\:{X}_{\mathrm{2,3}}&\:...&\:{X}_{2,d}\\\:{X}_{\mathrm{3,1}}&\:{X}_{\mathrm{3,2}}&\:{X}_{\mathrm{3,3}}&\:...&\:{X}_{3,d}\\\:...&\:...&\:...&\:...&\:...\\\:{X}_{m,1}&\:{X}_{m,2}&\:{X}_{m,3}&\:...&\:{X}_{m,d}\\\:&\:&\:&\:&\:\end{array}\right]$$

9 $$\:Y=\left[\begin{array}{lllll}{Y}_{\mathrm{1,1}}&\:{Y}_{\mathrm{1,2}}&\:{Y}_{\mathrm{1,3}}&\:...&\:{Y}_{1,d}\\\:{Y}_{\mathrm{2,1}}&\:{Y}_{\mathrm{2,2}}&\:{Y}_{\mathrm{2,3}}&\:...&\:{Y}_{2,d}\\\:{Y}_{\mathrm{3,1}}&\:{Y}_{\mathrm{3,2}}&\:{Y}_{\mathrm{3,3}}&\:...&\:{Y}_{3,d}\\\:...&\:...&\:...&\:...&\:...\\\:{Y}_{m,1}&\:{Y}_{m,2}&\:{Y}_{m,3}&\:...&\:{Y}_{m,d}\\\:&\:&\:&\:&\:\end{array}\right]$$

10 $$\:h=\left[\begin{array}{l}{h}_{1}({X}_{\mathrm{1,1}},{X}_{\mathrm{1,2}},{X}_{\mathrm{1,3}},...,{X}_{1,d})\\\:{h}_{2}({X}_{\mathrm{2,1}},{X}_{\mathrm{2,2}},{X}_{\mathrm{2,3}},...,{X}_{2,d})\\\:{h}_{3}({X}_{\mathrm{3,1}},{X}_{\mathrm{3,2}},{X}_{\mathrm{3,3}},...,{X}_{3,d})\\\:...\\\:{h}_{m}({X}_{m,1},{X}_{m,2},{X}_{m,3},...,{X}_{m,d})\\\:\end{array}\right]$$

Mothers excel in fitness among all bird species because they instruct their young on obtaining food and survival. During the study, the optimal location, labeled $\:{X}_{best}$, undergoes revisions. Offspring of mothers, referred to as normal birds ($\:{\mathrm{X}}_{\mathrm{n}\mathrm{d}}$), exhibit devoted behavior. $\:{X}_{Gbest}$ represents the most optimal solution discovered during the search process. By incorporating the given equations to account for time and population migration, the optimizer leverages the DTO approach to track the movements of swimming birds.

The position update follows two strategies: attraction toward the best position (Eq. 11) or direct velocity-based movement (Eq. 12), selected probabilistically as shown in Eq. (13). Specifically, if the random value r₃ < 0.5, the dipper moves toward an attractive position A; otherwise, it follows the velocity-based position B. The velocity update mechanism, presented in Eq. (14), integrates inertia, cognitive, and social components to balance exploration and exploitation.

11 $$\:A={X}_{best}\left(i\right)-{K}_{1}.|{K}_{2}.{X}_{best}(i)-X(i\left)\right|$$

12 $$\:B=X\left(i\right)+Y(i+1)$$

13 $$\:\begin{array}{c}X(i+1)=\left\{\begin{array}{ll}A&\:\mathrm{i}\mathrm{f}{r}_{3}<0.5\\\:B&\:otherwise\end{array}\right.\end{array}$$

14 $$\:\begin{array}{ll}Y(i+1)&\:={K}_{3}Y\left(i\right)+{K}_{4}{r}_{1}\left({X}_{best}\right(i)-X(i\left)\right)+{K}_{5}{r}_{2}({X}_{Gbest}-X(i\left)\right)\end{array}$$

For each iteration $\:i$, the best position of the birds is denoted by $\:{X}_{best}\left(i\right)$, the average position of the birds is denoted by $\:X\left(i\right)$, and the speed of the birds on iteration $\:i+1)$ is marked by $\:Y(i+1)$. Weight values $\:{K}_{1}$, $\:{K}_{2}$, and $\:{K}_{3}$ are chosen dynamically from the range [0–2] during optimization; constant values $\:{K}_{4}$ and $\:{K}_{5}$ are 1.7 and 1.8, respectively. The values of $\:{r}_{1}$, $\:{r}_{2}$, and $\:{r}_{3}$ are all random integers in the range $\:\left[\mathrm{0,1}\right]$.

#### Particle swarm optimization (PSO)

The PSO algorithmis based on the mechanics of schools or flocks of, say, fish or birds. A particle swarm is employed collaboratively to model a complex search space and obtain an accurate solution for the given problem [31]. Giving establishes this balance by adding new velocity to a particle; the new velocity depends on the velocity from the previous iteration and the attraction to the local and global best. The PSO algorithm operates on a swarm of N particles, where each particle has a position vector X and a velocity vector Y. In addition, it keeps a personal best position vector Xbest, signifying the particle’s best-found solution so far, and a global best position vector XGbest, defined as the overall best solution reached by the entire swarm. At each iteration t, each particle’s position and velocity will be updated according to the specific equations. The change of velocity depending on the position is described in Eq. (15):

15 $$\:Y(t+1)=w\mathrm{*}Y\left(t\right)+{c}_{1}\mathrm{*}{r}_{1}\mathrm{*}({X}_{best}-X(t\left)\right)+{c}_{2}\mathrm{*}{r}_{2}\mathrm{*}({X}_{Gbest}-X(t\left)\right)$$

A particle’s inertia weight (w) controls the effect of its prior velocity on its current velocity, while acceleration coefficients determine the impact of the best local and best global solutions on the particle’s velocity ($\:{c}_{1}$ and $\:{c}_{2}$). The method additionally considers two random values, $\:{r}_{1}$, and $\:{r}_{2}$, which must be between $\:0$ and $\:1$. The position is updated using Eq. (16):

16 $$\:X(t+1)=X\left(t\right)+Y(t+1)$$

When a modified position yields a better value for the objective function than the last, the best value is replaced by it if it is the optimal solution. Exploring the space composed of all the possible values of vector X is a source of updates to the best global solution as soon as there is an improvement in the current optimal solution for any particle. On the contrary, PSO continues iterating until an end condition is met.

### Source separation methods

#### Blind source separation (BSS)

Blind Source Separation (BSS) is a complex signal processing approach that aims to separate a single signal contaminated by an unobserved mixing process into its sources without requiring any additional information [32]. In real-world applications where data collected from sensors or data streams is a combination of multiple underlying sources, the signals are often highly complex. BSS serves as an effective tool for dissecting this level of complexity, which often confuses experts, by identifying the contributions of sources and solving problems. The application is not confined to boundaries, allowing for interdisciplinary domains such as telecommunications, signal processing, audio processing, biomedical engineering, and image processing. The details of this algorithm are as follows:

BSS operates by decomposing the observed mixed signals X into their constituent sources S, under the assumption of statistical independence, using a linear mixing model as expressed in Eq. (24):

24 $$\:X=AS+N$$

Where:

- X represents an $\:n\times\:p$ matrix, indicating the observed mixed signals.
- $\:S$ represents an $\:n\times\:p$ matrix, indicating the underlying independent sources.
- $\:A$ denotes a $\:p\times\:p$ mixing matrix that represents the linear transformations applied to the sources.
- $\:N$ denotes an $\:n\times\:p$ matrix accounting for additive noise.

The primary objective of BSS is to estimate the unmixing matrix W, which can recover the original sources S from the observed mixed signals X by solving the inverse problem as shown in Eq. (25):

25 $$\:S=WX$$

The unmixing matrix $\:W$ is designed to optimize the statistical independence of the estimated sources.

BSS is a key element because it prevents noise from increasing, which is usually the result of fusing mixed signals back to their sources. Noise sources in recorded, mixed signals are generally correlated and have identical statistical properties. As a result, noise and signals in the observed mixed signals cannot be separated. Under the BSS, independent estimating statistics improve the separation of signal components from noise components. Therefore, by using this filtering, a more polished, noise-reduced representation of the data is achieved, allowing experts to analyze desired features better and reduce potential interference from noise or unwanted effects.

Noise suppression is pivotal in super-resolution reconstruction, as it leverages statistical independence to separate noise from the signal. As sounds will present repetitions and similarities with signal parameters, they usually end up being associated with these aspects of the noise-period-containing mixes. When applied, BSS accurately estimates noise from statistically independent directions, which is necessary for recovering the clean signal. This processing results in a more precise representation of the data, thereby enabling practical interpretation and analysis of the generated information that can be remembered.

The effectiveness of a BSS algorithm in reducing noise can be assessed using three key metrics. Source-to-Distortion Ratio (SDR) measures the power of estimated sources relative to the power of separation artifacts. Source-to-Interference Ratio (SIR) quantifies the separation capability by comparing desired source power to interference power. Source-to-Artifact Ratio (SAR) evaluates the power of artifacts introduced during the separation process.

#### Principal component analysis (PCA)

PCA is a useful statistical tool for reducing dimensionality and exploring variables in multivariate data analysis. In many real-world applications worldwide, data sources typically include many variables, making it challenging to bring the data into view and analyze them properly [33]. PCA addresses this issue by projecting the original dataset onto a new set of variables, the principal components, which represent significant variations in the data. This way, by retaining most of the variance in the data set while reducing its dimensionality, PCA facilitates the discovery of the model’s underlying structure, helps identify the most informative features, and brings the most relevant information to light.

PCA begins with a dataset X consisting of n observations (rows) and p variables (columns). Each observation represents a point in a p-dimensional space, where each variable corresponds to a different dimension. Centroiding data means subtracting each variable’s average from its values, as shown in Eq. (17). It ensures that the principal components are centered on the coordinate system’s centroid and are used for all subsequent calculations, guaranteeing that the data represent their variability rather than their values.

17 $$\:{X}_{\mathrm{centered}}=X-\stackrel{⃐}{X}$$

The covariance matrix Σ summarizes the pairwise relationships between variables in the centered data, as computed in Eq. (18). Each element Σ_i_ⱼ represents the covariance between variables i and j, indicating how they co-vary with each other.

18 $$\:{\Sigma\:}=\frac{1}{n}\left({X}_{\mathrm{centered}}{)}^{T}\right({X}_{\mathrm{centered}})$$

PCA computes the eigenvalues (λ₁, λ₂,…, λₚ) and corresponding eigenvectors (v₁, v₂,…, vₚ) of the covariance matrix Σ. Eigenvalues represent the amount of variance explained by each principal component, while eigenvectors represent the directions (axes) of maximum variance in the data, as expressed in Eq. (19).

19 $$\:{\Sigma\:}{\mathrm{v}}_{i}={\lambda\:}_{i}{\mathrm{v}}_{i}$$

PCA selects the top $\:k$ eigenvectors associated with the largest eigenvalues as the principal components. These eigenvectors capture the most significant patterns of variation in the data and are arranged in descending order of importance. Then, PCA is the technique used to transform the original variables into elements of the new subspace by utilizing the principal components derived from the process. Every observation is labeled by a pair of its coordinate values along the principal components, which are the projections of the data matrix onto its set of eigenvectors, as shown in Eq. (20).

20 $$\:\text{Projected Data}={X}_{\mathrm{centered}}\cdot\:{V}_{k}$$

Where $\:{V}_{k}$ is the matrix containing the top $\:k$ eigenvectors as columns.

The principal component loadings can be interpreted in terms of the variables by studying the scores of each variable on the principal components that represent coefficients. A high load factor indicates a fractional share of the total variance, and a negative value points to a negative contribution to a significant component. The loadings are calculated using Eq. (21).

21 $$\:\mathrm{Loadings}=\mathrm{Eigenvectors}\times\:\sqrt{\mathrm{Eigenvalues}}$$

PCA precisely addresses the problem of including noise in the data. PCA makes this possible by maximizing the information provided and stripping away the rest, creating a clear boundary between the signal (meaningful patterns) and noise (random variations). The first segment, containing the largest eigenvalues, represents significant patterns in the data, while the components with smaller eigenvalues exhibit noise or insignificant variation. In this way, dimensionality reduction is achieved through PCA by deleting less important elements, or noise, that do not provide meaningful insight into the data representation.

#### Independent component analysis (ICA)

Independent component analysis (ICA) is a multivariate statistical method that was developed to decompose observed quantities into carefully defined independent components [34]. For example, changes in the intensity of signals detected by sensors or documented in data streams often result from combining so many underlying sources that it is hard to disentangle them. ICA with-sculpts this problem of embodiment by decomposing the actual data into its base components, whose statistical independence is presumed. This feature allows ICA to mainly perform blind source separation, denoising, and feature extraction across diverse areas, such as signal processing, neuroscience, finance, and image processing.

Independent Component Analysis (ICA) is fundamentally based on two core assumptions: statistical independence and non-Gaussianity. The assumption of statistical independence posits that the underlying source signals are mutually independent, meaning that the presence of the others does not affect the probability distributions of the sources. In addition, ICA requires that the source signals exhibit non-Gaussian distributions. This requirement is critical because purely Gaussian signals cannot be effectively separated using independence criteria alone, as their statistical properties do not provide sufficient information for unique source identification.

ICA, besides its critical role in noise reduction in data, can also split mixed signals into independent prototypes. On the contrary, the noise components are highly coupled and share the same statistical properties, so they fall into a single block in the motorized sources. Mathematically, information theory uses the independence maximization principle; therefore, independent component analysis (ICA) can be used to separate signal and noise components with greater representational capacity for the data. This detachment from the data allows the researchers to analyze only the meaningful features. This, in turn, will enable them to minimize the effect of noise and unwanted anomalies.

### Performance evaluation metrics

The peak signal-to-noise ratio (PSNR) and normalized cross-correlation (NC) [35,36] values are frequently used to evaluate algorithms. When comparing an image’s watermarked and unwatermarked versions, the PSNR and NC can be calculated using Eq. (26):

26 $$\:MSE=\frac{1}{M\times\:N}\sum\:_{i=0}^{M-1}\sum\:_{j=0}^{N-1}{\left[I(i,j)-{I}_{w}(i,j)\right]}^{2}$$

where $\:{I}_{w}(i,j)$ is watermarked image and $\:I(i,j)$ is the original image. The Peak Signal-to-Noise Ratio (PSNR) is calculated as shown in Eq. (27):

27 $$\:PSNR=10lo{g}_{10}\left(\frac{25{5}^{2}}{MSE}\right)$$

The Normalized Cross-Correlation (NCC) between the original and extracted watermarks is determined using Eq. (28):

28 $$\:NCC=\frac{\sum\:_{i=0}^{M-1}\sum\:_{j=0}^{N-1} W(i,j)\widehat{W}(i,j)}{\sqrt{\sum\:_{i=0}^{M-1} \sum\:_{j=0}^{N-1}W(i,j)^{2}}\sqrt{\sum\:_{i=0}^{M-1}\sum\:_{j=0}^{N-1}\widehat{W}(i,j)^{2}}}$$

where $\:W(i,j)$ represents watermarked picture and $\:\widehat{W}(i,j)$ represents a watermarked image after one of the attacks has been performed. NCC and PSNR are used to create fitness functions to make the algorithm as secure and stealthy as possible. The fitness function is formulated as expressed in Eq. (29):

29 $$\:fit\left(\alpha\:\right)=\alpha\:\frac{PSNR}{\beta\:}+(1-\alpha\:)\frac{1}{n}\sum\:_{i=1}^{n}\mathrm{‍}N{C}_{i}$$

$\:W$here the scaling factor is denoted by $\:\alpha\:$, $\:\beta\:$ is a constant used to modify the magnitude of the $\:PSNR$ (in this work, $\:\beta\:$ is chosen as 37 dB), and n is the number of attacks.

In addition, the intensity fidelity (IF) criterion is included in the experiments conducted to assess the performance of the proposed methodology, as defined in Eq. (30):

30 $$\:IF=1-\frac{\sum\:_{i,j} I(i,j)-{I}_{w}(i,j{)}^{2}}{\sum\:_{i,j}I(i,j)\times\:{I}_{w}(i,j)}$$

## Proposed methodology

This section presents a comprehensive description of the proposed watermarking framework, integrating hybrid transform-domain techniques with nature-inspired optimization algorithms and advanced noise-reduction methods. The methodology unfolds systematically: first, the novel Dipper-Throated Particle Swarm Optimization (DTPSO) algorithm is introduced as the core optimization mechanism; second, the watermark embedding procedure using DWT-DCT hybrid transforms is detailed; third, the extraction algorithm for blind watermark recovery is presented; and finally, the noise reduction pipeline incorporating AlexNet-based feature extraction combined with optimized BSS techniques is thoroughly explained.

### Feature extraction and noise reduction

The proposed methodology begins with AlexNet, a pre-trained convolutional neural network that transforms images into numerical representations through hierarchical layers. This deep learning architecture extracts both low-level details and high-level semantic features, converting raw pixel values into abstract numerical data that captures essential image characteristics while discarding irrelevant information, thereby providing the foundation for subsequent noise-reduction processing.

Blind Source Separation serves as the comprehensive framework for noise reduction, representing an advanced signal processing paradigm that decomposes mixed signals into their sources without prior knowledge of the mixing mechanism. BSS operates on the premise that observed signals are linear combinations of independent or non-Gaussian source signals, and employs statistical analysis to estimate and recover the sources effectively. This framework encompasses various methodologies that separate noise from valuable image features, making it effective at handling distortions caused by sensor errors, compression artifacts, and transmission imperfections.

Within the BSS framework, Principal Component Analysis serves as a variance-based method that processes AlexNet-extracted features via normalization, covariance matrix computation, and eigenvalue decomposition. By projecting data into a lower-dimensional space, PCA eliminates noise while retaining essential structural information, enhancing watermark robustness against compression, resizing, and filtering attacks. Independent Component Analysis provides an alternative BSS implementation based on statistical independence principles, processing numerical features through linear transformations to distinguish pure signals from noise contamination. This approach maintains key image features while reducing noise, thereby enhancing watermark security and resilience against adversarial attacks.

To enhance BSS performance, optimization techniques including Dipper-Throated Particle Swarm Optimization (DTPSO), Dipper-Throated Optimization (DTO), and Particle Swarm Optimization (PSO) are employed. DTPSO-BSS integrates PSO with the Dipper-Throated technique to optimize parameter settings, where particle swarms represent possible solutions and adaptively adjust threshold values based on image characteristics and noise patterns. DTO-BSS leverages adaptive threshold adjustments that respond to intrinsic image properties, while PSO-BSS combines collective swarm intelligence to discover optimal configurations for noise-feature separation. These optimization methods strengthen the watermarking system by enhancing noise suppression capabilities and ensuring robust performance across diverse image datasets and noise types.

### The proposed DTO + PSO algorithm

#### Algorithm 1

goes through the mechanism of the proposed hybrid optimization algorithm. The basis population of this algorithm is a set of vectors, which are the values of the scaling factors. In addition, these vectors would also represent the PSNR and NCC values obtained as the corresponding measurements. Instead, it provides almost the best scalable performance when evaluated using the PSNR and NCC criteria.

**Figure:**
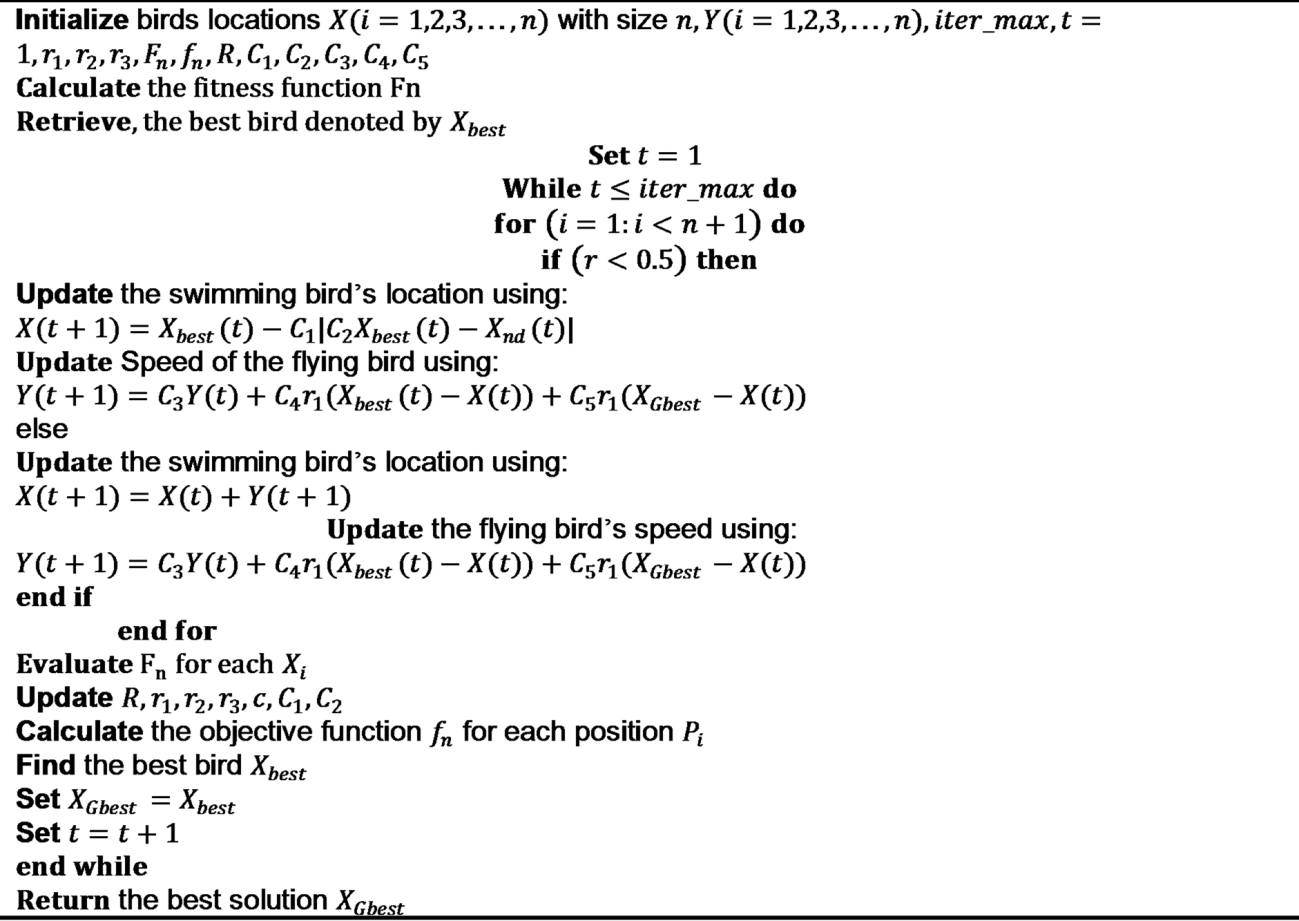
Algorithm 1 : The proposed DTPSO algorithm.

#### Algorithm 1

The proposed DTPSO algorithm.

### Watermark embedding algorithm

The Down Carrier Transformation (DCT) and the Discrete Wavelet Transform (DWT) methods are widely used in generating digital watermarks. In contrast to the DCT, the DWT can analyze the signal in both the temporal and frequency domains via mathematical operations that transform it from the spatial to the frequency domain. The high frequency in both DWT and DCT coefficients, which play a prominent role in determining the optimal location for watermark embedding without loss of quality, is a relevant factor. The watermark uses a combination of DCT and DWT. Thus, it becomes difficult to forge and merge with other data to the extent that altering or deleting the watermark becomes impossible. The effectiveness of this technique lies in its ability to transform authorized users into those who can discreetly extract the watermark while the media is in use.

**Fig. 3:**
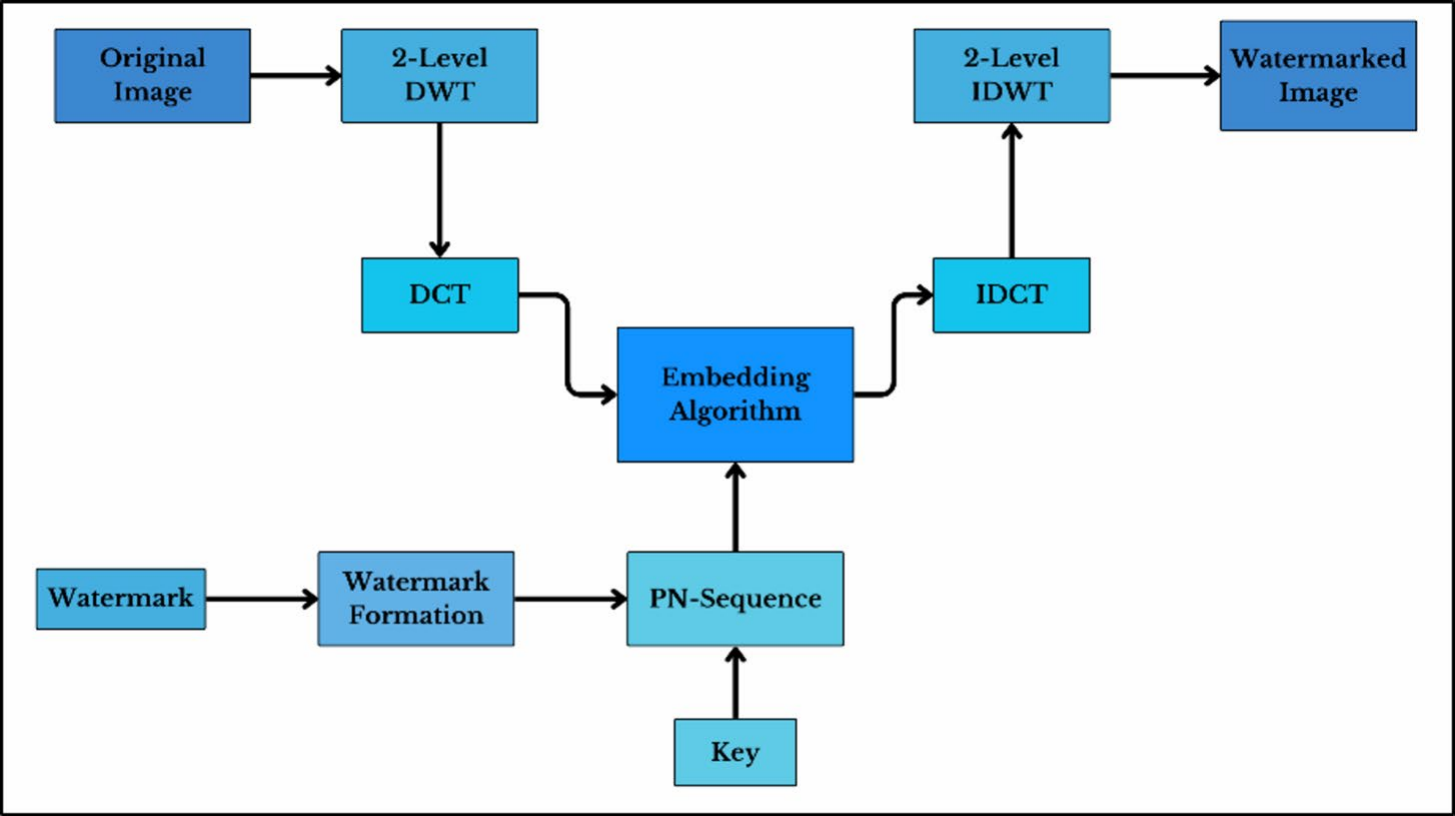
Watermark Embedding Process Using Hybrid DWT-DCT Transform.

Figure 3 presents the steps of the watermark extraction. The benefit of the combined DWT-DCT method in watermarking is that it operates as a blind watermarking technique, eliminating the need to transmit the host image to extract the embedded watermark.

### Watermark exacting algorithm

In digital watermarking, watermark extraction. Removal of the watermark is an inevitable part of the process for the media that an engineer has watermarked. Reflecting its high security and ability to withstand multiple attackers, the watermark extraction algorithm based on DWT + DCT is widely used. This approach splits the image into multiple frequency bands using a discrete wavelet transform (DWT). Next, the discrete cosine transforms (DCT) are applied to each image band separately. The next step is to check the DWT and DCT high-frequency coefficients against those used as watermarks.

**Fig. 4:**
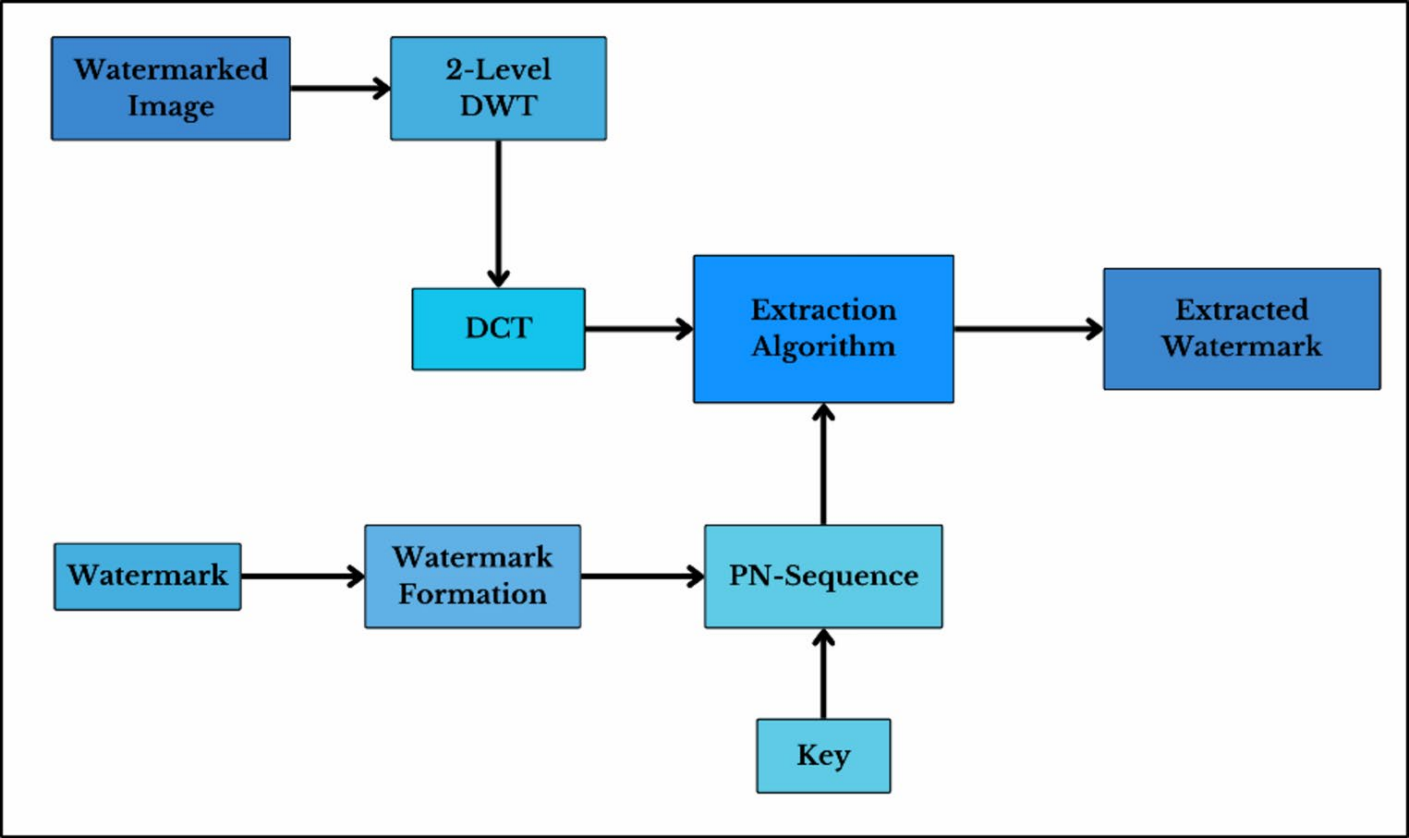
Watermark Extraction Process Using Hybrid DWT-DCT Transform.

The watermark in the coefficients is subtracted from the original coefficients during decoding. Therefore, it will operate the instruction on the embedding. Copyright protection, content verification, and data hiding, among many other applications, are only a few of the things the DWT + DCT-based watermark extraction technique can do, as it provides precise results and is immune to features such as noise addition, filtering, compression, and geometric distortion. Figure 4 shows how watermarks can be extracted.

## Experimental results

Table 2 presents what we achieved in the experiment. In interpreting this data, the alternative procedures should be compared with this method. During the advanced feature-matching phase, a difference-optimizing algorithm adjusts the scaling factor and extracts a criterion used for message extraction.

**Table 2:** Performance Metrics of Embedded Image Extraction Across Different Methods.

	DFT+ SVD	DWT+ DCT	DWT+ DCT+ BFO	DWT+ DCT+ GOA	DWT+ DCT+ PSO	DWT+ DCT+ DTO	DWT+ DCT+ DTPSO
PSNR	44.75	55.75	60.21	61.32	62.44	64.11	65.78
NCC	0.659	0.799	0.839	0.8629	0.8789	0.8949	**0.9189**
IF	0.8646	0.8646	0.8646	0.8646	0.8646	0.8646	**0.8646**
BER	0.198	0.128	0.0931	0.0757	0.0583	0.0408	**0.0234**
Accuracy	0.802	0.872	0.9069	0.9243	0.9417	0.9592	**0.9766**

As shown in Table 2, the proposed DWT + DCT+DTPSO method achieved the highest PSNR of 65.78 dB, the highest NCC of 0.9189, the lowest BER of 0.0234, and the highest accuracy of 0.9766 among all compared methods. These results demonstrate that the proposed model provides the most favorable trade-off between watermark imperceptibility, extraction reliability, and overall robustness. Although the obtained NCC value of 0.9189 does not reach the ideal value of 1, it is still considered satisfactory in the context of the present study because it is the highest among all compared methods. Moreover, this result is supported by the lowest BER and the highest accuracy, confirming that the extracted watermark maintains strong similarity with the original watermark and can be recovered reliably. Nevertheless, the NCC value indicates that there is still room for further enhancement in correlation-based watermark recovery under more challenging attack scenarios.

**Fig. 5:**
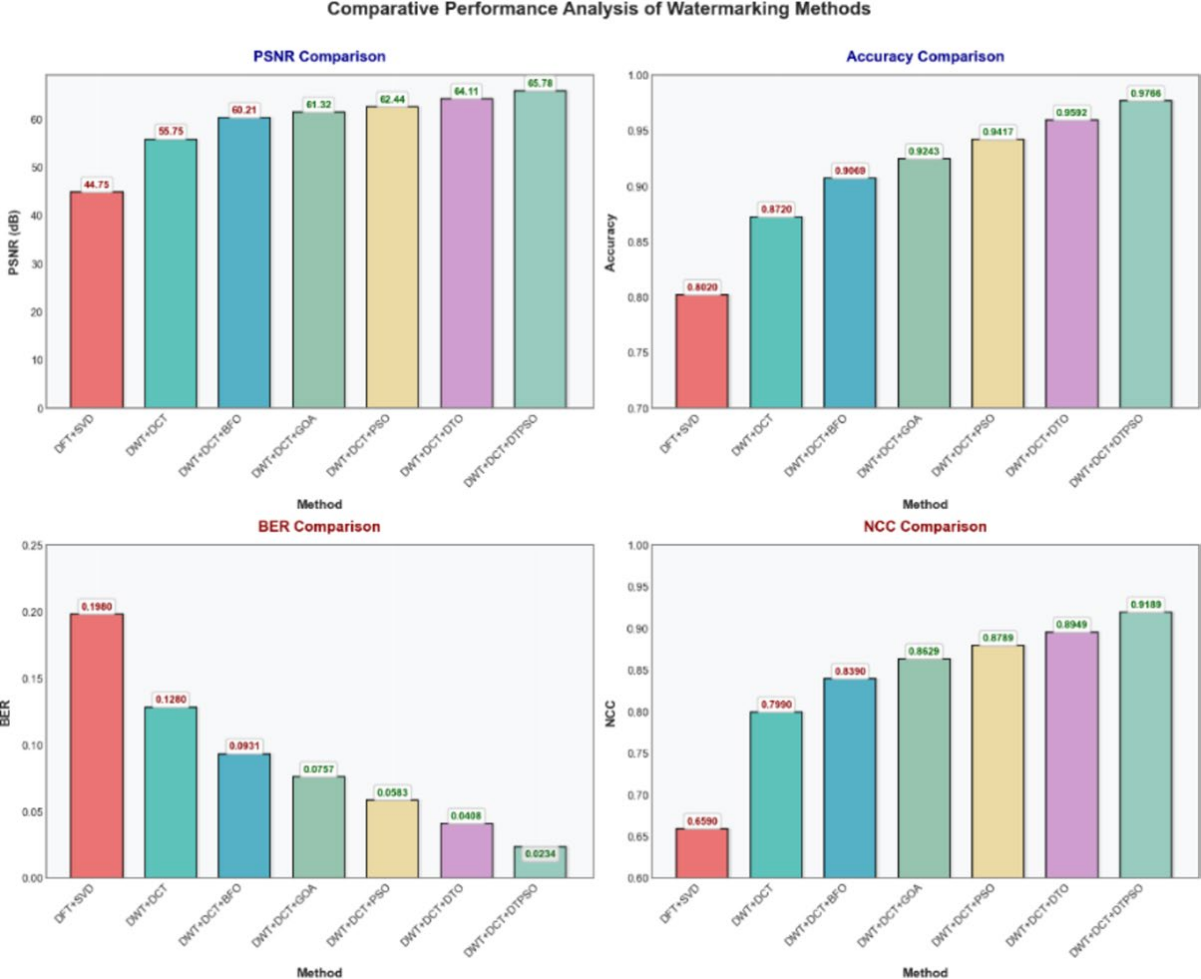
Comparative performance analysis of digital watermarking methods on color image.

Figure 5 reveals a comparative performance evaluation of six digital watermarking methods – DFT-SVD, DWT-DCT, DWT-DCT + BFO, DWT-DCT + GDA, DWT-DCT + PSO, and DWT-DCT + DTO – assessed using Peak Signal-to-Noise Ratio (PSNR), Accuracy, Bit Error Rate (BER), and Normalized Correlation Coefficient (NCC). The results demonstrate a consistent trend of improvement as methods evolve from DFT-SVD towards DWT-DCT + DTO, with the latter consistently achieving the highest values across all metrics.

DWT-DCT + DTO exhibits a peak PSNR of 65.78 dB, an accuracy of 0.9766, a low BER of 0.0024, and a strong NCC of 0.9189. Conversely, DFT-SVD performs worst, with a PSNR of 44.75 dB, an accuracy of 0.8020, a high BER of 0.1980, and an NCC of 0.6590. The addition of optimization algorithms – BFO, GDA, PSO, and DTO – to the DWT-DCT base method demonstrably enhances performance across all cases. This suggests that DWT-DCT provides a robust foundation, further strengthened by these optimization techniques. Consequently, the data strongly support the conclusion that DWT-DCT + DTO is the most effective watermarking technique tested, offering the best balance of image quality preservation and watermark robustness.

**Fig. 6:**
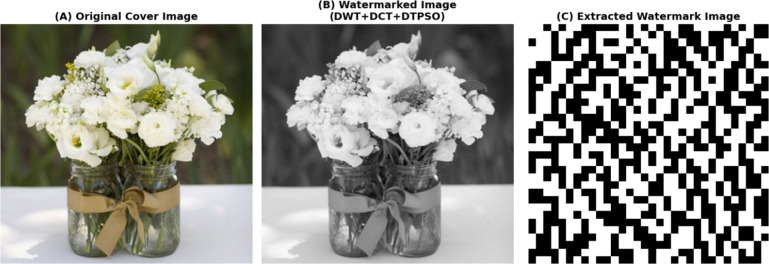
Comparative performance analysis of digital watermarking methods on color image.

Figure 6 presents the visual outcome of the proposed watermarking framework based on DWT + DCT+DTPSO. As observed in Fig. 6 (A), the original cover image serves as the input image for the embedding process. Figure 6 (B) illustrates the generated watermarked image, reflecting the imperceptibility of the proposed approach. Finally, Fig. 6 (C) depicts the extracted watermark image, confirming the robustness and effectiveness of the proposed method in preserving and recovering the embedded watermark.

Table 3 shows the results of the variance analysis, and, based on the information in the table, a p-value lower than 0.005 indicates statistical significance between the proposed strategy and the alternative procedures that were part of the experiments analyzed.

**Table 3:** ANOVA statistical analysis results for embedded image extraction across different methods.

	SS	DF	MS	F (DFn, DFd)	*P*-value
Treatment	10,482	6	1747	F (6, 63) = 1609	*P* < 0.0001
Residual	68.41	63	1.086		
Total	10,551	69			

Table 4 demonstrates the statistical significance of the proposed DTPSO method through the Wilcoxon signed-rank test analysis across seven watermarking approaches. The test compares each method’s performance against a theoretical median of zero, with all approaches yielding highly significant p-values of 0.002 (*p* < 0.005), confirming substantial improvements over baseline performance. Notably, the median values increase progressively from 35.18 for DFT + SVD to 77.91 for the proposed DWT + DCT+DTPSO method.

**Table 4:** Statistical validation of watermarking methods using wilcoxon signed-rank test analysis.

Theoretical median	DFT + SVD	DWT + DCT	DWT + DCT+BFO	DWT + DCT+GOA	DWT + DCT+PSO	DWT + DCT+DTO	DWT + DCT+DTPSO
	0	0	0	0	0	0	0
Sum of -ve ranks	0	0	0	0	0	0	**0**
Sum of +ve ranks	55	55	55	55	55	55	**55**
Actual median	35.18	44.67	49.66	47.56	51.27	57.78	**77.91**
Sum of ± ranks	55	55	55	55	55	55	**55**
Discrepancy	35.18	44.67	49.66	47.56	51.27	57.78	**77.91**
Two-tailed (P-value)	0.002	0.002	0.002	0.002	0.002	0.002	**0.002**
Number of values	10	10	10	10	10	10	**10**

**Fig. 7:**
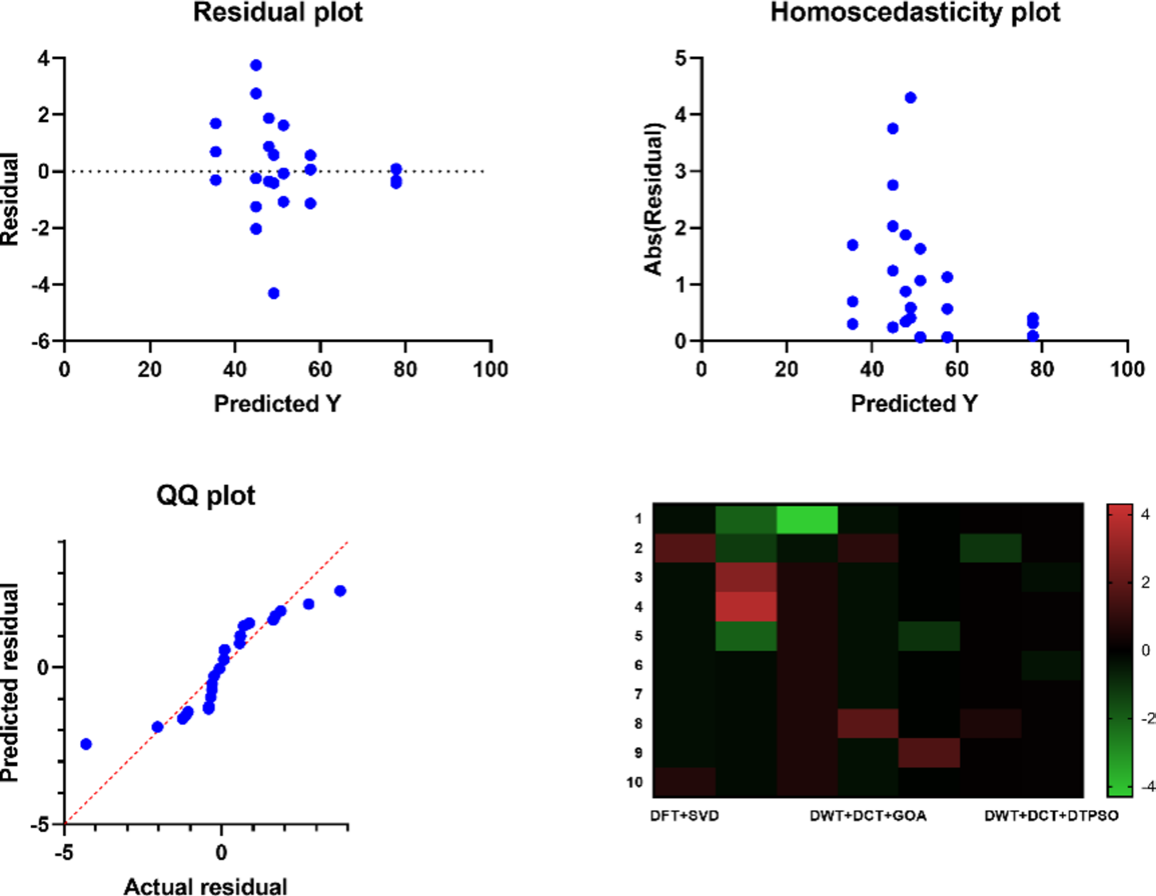
Statistical Validation Plots: QQ Plot, Residual Plot, Homoscedasticity Plot, and Correlation Heatmap.

Figure 7 presents a heatmap, QQ plot, residual plot, and homoscedasticity plot, collectively validating the statistical robustness of the proposed watermarking method. The QQ plot demonstrates normal distribution of residuals, the residual plot shows randomly scattered points around zero, indicating no systematic bias, the homoscedasticity plot reveals consistent variance across predicted values, and the correlation heatmap illustrates distinct performance characteristics of DWT + DCT+DTPSO compared to traditional methods, providing comprehensive statistical evidence for the reliability and effectiveness of the proposed DTPSO-BSS approach.

**Fig. 8:**
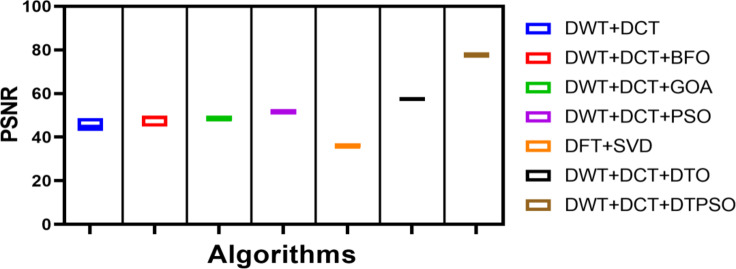
The PSNR achieved by the proposed algorithm compared to other algorithms.

Figure 8 shows that digital watermarking using the PSNR (Peak Signal-to-Noise Ratio) is a very viable approach for image quality assessment. PSNR histograms, a staple tool in watermarking, serve as a guide for analyzing image distortion and compression artifacts across versions. The watermark is worn over time, as the histogram can preserve the PSNR threshold and yield very few decompression artifacts. PSNR histograms provide prompt feedback on image quality loss across watermarking algorithms, enabling optimization of image compression levels while ensuring image integrity, thereby enhancing image quality and preventing distortion in watermarking systems.

**Fig. 9:**
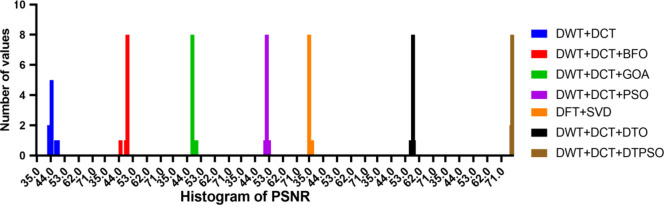
PSNR performance comparison histogram across optimization methods.

The PSNR histogram in Fig. 9 underscores the importance of these tools, which are widely used in watermarking systems to assess image quality and levels of distortion. PSNR histogram analysis is a prerequisite for evaluating the quality of lossy compression and artifacts introduced during image watermarking; thus, the PSNR values must be high enough to maintain image quality. The histograms not only facilitate a clear visual scan of image results but also ensure quality control and disclose the specific watermarking methods used to maintain image fidelity.

Table 5 Presents a comparative analysis of the computational performance of the investigated watermarking methods in terms of execution time, CPU utilization, GPU utilization, and memory consumption. These metrics provide important insight into the practical efficiency of each method in addition to watermarking accuracy and robustness. As observed, the proposed DWT + DCT+DTPSO approach demonstrates the best overall computational efficiency by achieving the shortest execution time of 46.5 s and the lowest memory requirement of 768 MB, indicating its suitability for efficient and resource-aware watermarking applications.

**Table 5:** Comparative computational performance of the investigated watermarking methods in terms of execution time, CPU usage, GPU usage, and memory consumption.

Method	Time (sec)	CPU Usage (%)	GPU Usage (%)	Memory (MB)
DFT + SVD	388.2	39	40	2048
DWT + DCT	294.7	38	90	1792
**DWT + DCT+BFO**	**211.7**	**28**	**31**	**1536**
**DWT + DCT+GOA**	**126.3**	**37**	**62**	**1280**
**DWT + DCT+PSO**	**81.6**	**69**	**68**	**1024**
**DWT + DCT+DTO**	**58.1**	**61**	**51**	**896**
**DWT + DCT+DTPSO**	**46.5**	**48**	**87**	**768**

The results presented in Table 5 clearly indicate substantial differences in computational efficiency among the examined watermarking techniques. The conventional DFT + SVD method exhibits the highest execution time at 388.2 s and the largest memory consumption at 2048 MB, reflecting its relatively high computational cost. In contrast, the hybrid transform-based and optimization-assisted methods show progressive improvement in efficiency. Among them, the proposed DWT + DCT+DTPSO model achieves the best performance, requiring only 46.5 s of execution time and 768 MB of memory, which are the lowest values among all compared methods. Although GPU usage for DWT + DCT+DTPSO reaches 87%, this higher utilization suggests more effective exploitation of hardware acceleration, which contributes to reducing overall processing time. These findings confirm that the proposed method not only improves watermarking effectiveness but also provides a computationally efficient solution suitable for real-time and large-scale image watermarking applications.

**Fig. 10:**
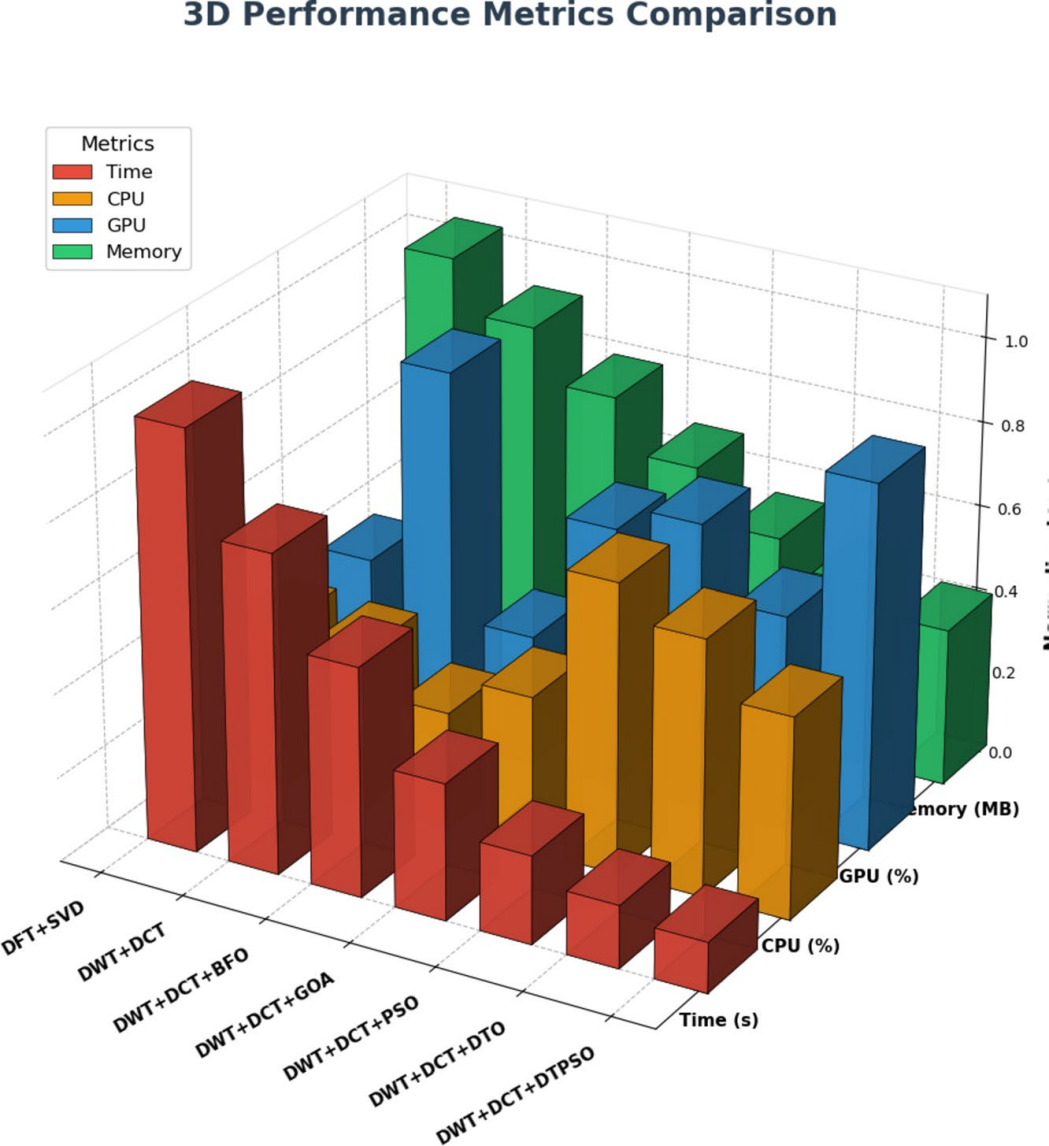
: Three-dimensional comparison of computational performance metrics for the investigated watermarking methods in terms of execution time, CPU usage, GPU usage, and memory consumption.

Figure 10 illustrates the comparative computational performance of the considered watermarking methods using a three-dimensional bar representation. As shown in Fig. 10, the execution time values reveal the relative speed of the examined methods, whereas the CPU usage values reflect processor engagement during computation. The GPU usage values further indicate the contribution of hardware acceleration to method implementation, and the memory values present the corresponding memory requirements of each technique. Collectively, Fig. 10 offers an overall assessment of computational resource consumption and highlights the superior efficiency of the proposed DWT + DCT+DTPSO model.

**Fig. 11:**
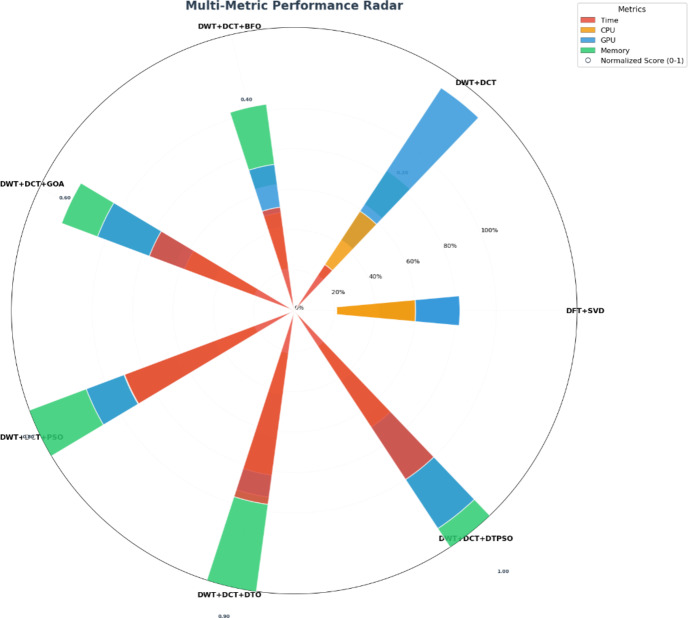
Multi-metric radar comparison of the investigated watermarking methods in terms of execution time, CPU usage, GPU usage, memory consumption, and normalized performance score.

Figure 11 presents a multi-metric radar visualization of the computational performance of the investigated watermarking methods. As illustrated in Fig. 11, the time metric reflects the relative execution speed of each method, while the CPU metric indicates the level of processor utilization during the watermarking process. The GPU metric represents the contribution of graphics processing resources to computational acceleration, whereas the memory metric describes the corresponding memory requirements of each technique. In addition, the normalized score shown in Fig. 11 provides an overall integrated assessment of computational efficiency by combining the individual performance indicators into a unified comparative measure. Overall, Fig. 11 offers a comprehensive view of the relative strengths and limitations of the examined methods and highlights the superior overall efficiency of the proposed DWT + DCT+DTPSO model.

**Fig. 12:**
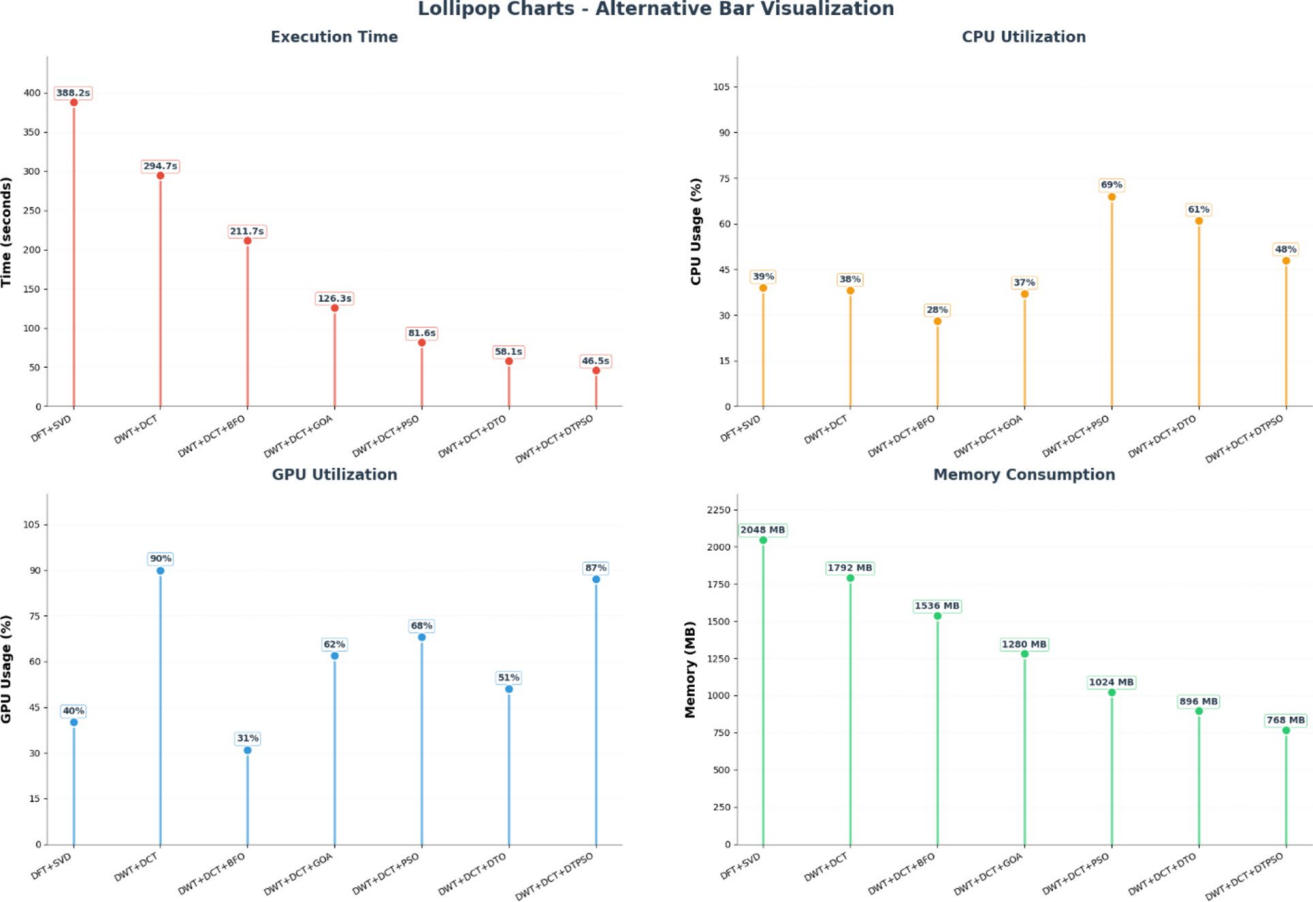
Lollipop-chart-based comparison of the investigated watermarking methods in terms of execution time, CPU utilization, GPU utilization, and memory consumption.

Figure 12 presents an alternative lollipop-chart visualization of the computational performance of the investigated watermarking methods. In Fig. 12, the execution time subplot illustrates the relative runtime required by each method, thereby highlighting differences in processing speed. The CPU utilization subplot in Fig. 12 shows the degree of processor engagement during the execution of each watermarking technique. Similarly, the GPU utilization subplot provides insight into the contribution of graphics processing resources to computational acceleration. In addition, the memory consumption subplot shown in Fig. 12 represents the memory requirements associated with each method. Collectively, Fig. 12 offers a clear and detailed visual comparison of computational resource utilization and further demonstrates the efficiency of the proposed DWT + DCT+DTPSO approach relative to the other benchmark methods.

### Linear regression test

Linear regression functions as an uncomplicated yet potent approach for evaluating and comparing digital image watermarking techniques. The performance of various methods can be evaluated using linear regression, which specifically measures how the proposed DWT + DCT+DTPSO algorithm compares with standard methods such as DFT + SVD, DWT + DCT, DWT + DCT+GOA, and DWT + DCT+PSO. The analysis measures the correlation between various performance metrics, including PSNR, SSIM, MSE, and embedding capacity, with each digital image watermarking technique. The application of linear regression allows researchers to evaluate the performance of the new method relative to existing methods.

Table 6 provides the outcomes of the linear regression model used to assess the tolerance of watermarking systems to rock universal patterns. In the table of F values, the variable that is used to show how methods are performing is introduced, and you will be able to note that the differences are significant between the methods. For instance, there is a massive difference in F values between DWT + DCT+PSO (0.02521) and DWT + DCT (0.812). DWT + DCT+PSO shows high performance, but DWT + DCT is the worst. Furthermore, the Fn and Fd degrees of freedom and p-value tabulations are added to the data table, providing detailed insight into the differentiation in performance among the various machine learning methods.

**Table 6:** The results of the linear regression test.

	DFT + SVD	DWT + DCT	DWT + DCT+ BFO	DWT + DCT+ GOA	DWT + DCT+ PSO	DWT + DCT+ DTO
F	0.457	0.812	0.332	0.471	0.025	0.059
P-value	0.005	0.004	0.006	0.005	0.009	**0.008**
DFn, DFd	1, 8	1, 8	1, 8	1, 8	1, 8	**1**,** 8**
Deviation from zero?	Crucial	Crucial	Crucial	Crucial	Crucial	**Crucial**

**Fig. 13:**
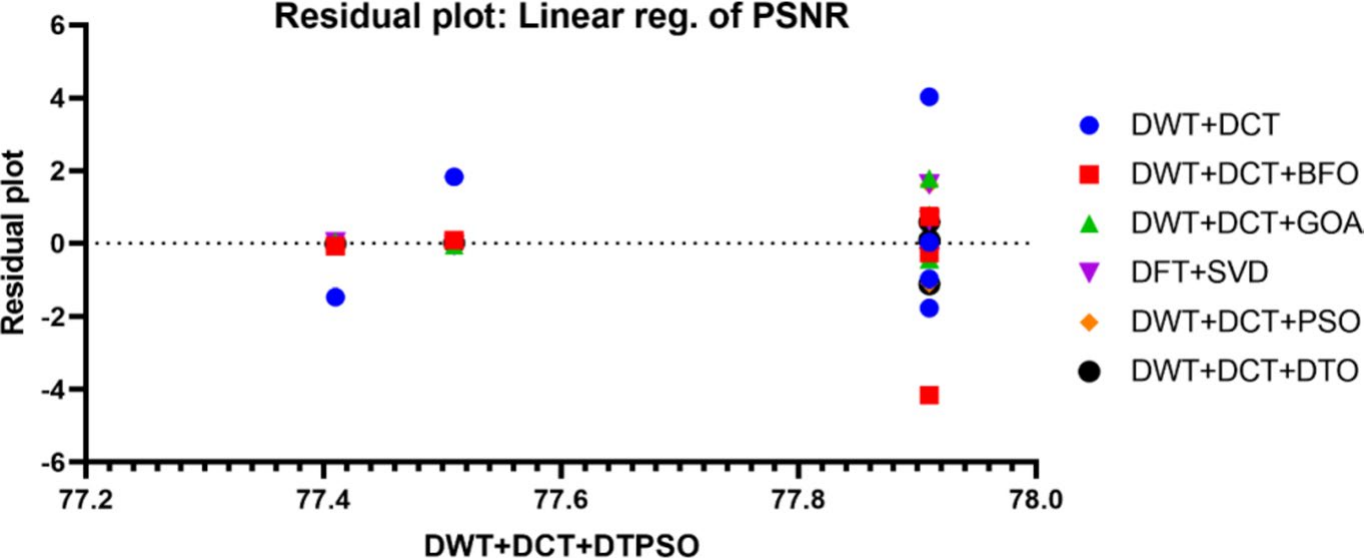
Linear regression analysis of optimization algorithm performance.

Figure 13 is a bar graph showing the objective function values obtained with different noise reduction algorithms, including PCA, ICA, BSS, PSO-BSS, DTPSO-BSS, and DTO-BSS. These objective function values are considered a numerical measure of each technique’s contribution to noise reduction and quality enhancement. This can be achieved by comparing the objective function values across all the optimization methods. In doing so, one can appreciate the effectiveness of each technique in optimizing the objective function operationally.

**Fig. 14:**
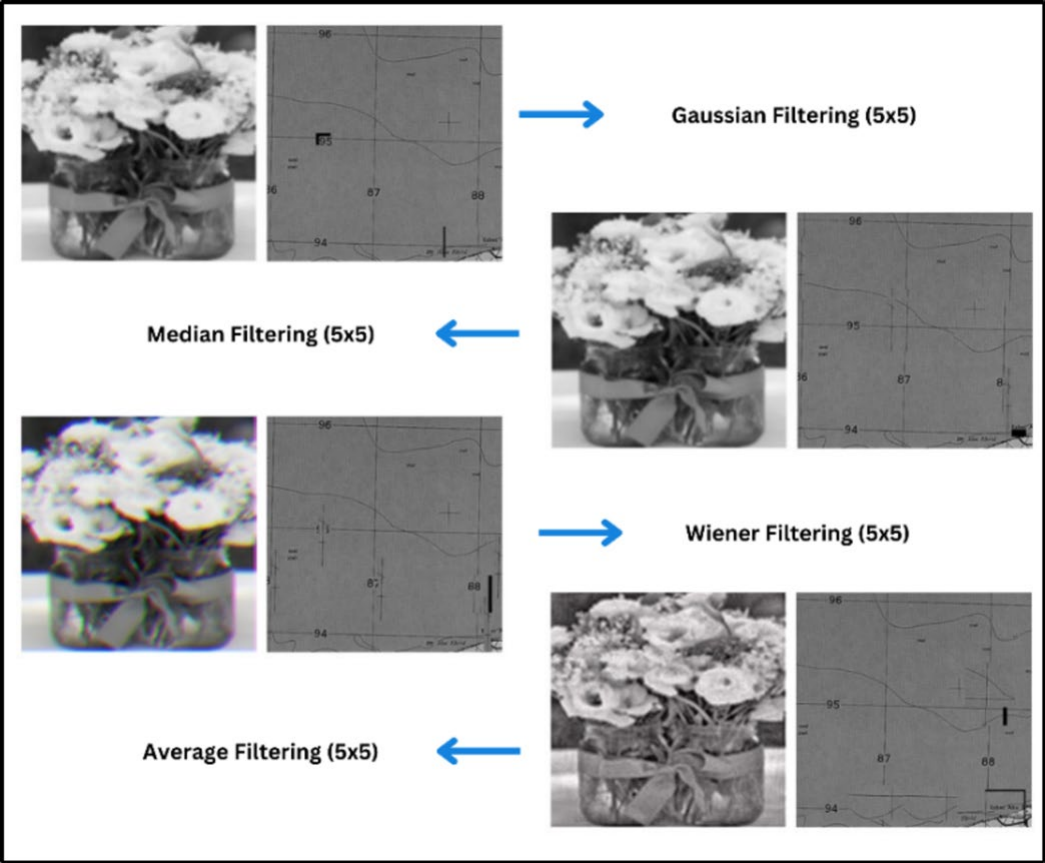
Map image recovery under different attack scenarios.

Another experimental area to study the proposed approach’s effectiveness when another watermark attack type is applied (a map file hidden in the flower image) was explored. This work targets all four attack types: Gaussian, median, Wiener, and average. The proposed method’s attack-specific map image recovery is illustrated in Fig. 14.

### Comparison with other methods

The proposed methodology is tested by running the quality histogram for the image subtraction process to study the best-fit method for image refinement. In our experiments, other methods are used for comparison. Table 7 shows the PSNR results for the restored map image hidden within the flower image. It can be seen that the DWT + DCT+DTPSO approach shows outstanding performance.

**Table 7:** PSNR Comparison of watermarking quality across multiple test images.

Image	Bees	ABC	K-NN	ACO	LGBA	DWT + DCT+DTPSO
Elaine	52.443	52.645	52.719	57.861	57.978	**75.648**
House1	51.431	51.993	51.973	55.667	56.062	**73.732**
Pirate	51.477	52.523	52.683	57.165	57.271	**74.941**
Boat	51.434	52.163	52.109	55.898	56.278	**73.948**
House2	53.007	56.749	56.805	60.992	61.302	**78.972**
Zelda	52.815	54.762	54.827	59.131	59.378	**77.048**

### Principal component analysis (PCA) results

Principal component analysis (PCA), the principal technique applied in watermarking, helps enhance the security of watermarked images by reducing distortion and ensuring high perceptual quality is maintained. Results from the PCA-based noise reduction process are summarized in Table 8. The SDR, ISR, and SAR metrics are detected for the first four components, PC1, PC2, PC3, and PC4, covering the most informative content.

**Table 8:** Noise Reduction performance using principal component analysis.

	PC1	PC2	PC3	PC4
SDR	3.261003	6.97937	8.211294	6.762345
ISR	5.357002	9.447814	12.57675	8.249709
SAR	11.67647	9.651535	8.546325	14.31798

### ICA (independent component analysis) results

This section presents the results of using ICA for noise removal. Table 9 presents the performance of the four independent components—ICA1 to ICA4—based on SDR, ISR, and SAR metrics, providing a clear view of each component’s performance.

**Table 9:** Noise reduction performance using independent component analysis.

	ICA1	ICA2	ICA3	ICA4
SDR	5.719003	9.43737	10.66929	9.220345
ISR	7.815002	11.90581	15.03475	10.70771
SAR	14.13447	12.10953	11.00433	16.77598

From the results, one can observe that the ICA method essentially reduces noise in the components, which in turn makes watermarked images more perceptually pleasing and robust. Importantly, each stripped-out subspace component produces significant improvements (in noise canceling metrics) like SDR, ISR, and SAR, demonstrating ICA efficiency in mitigating noise interference. The ICA3 component, in particular, performs best among the independent components and leaves even wider gaps in the other metrics evaluated. ICA extracts hidden information from image data and, at the same time, controls the noise level. Therefore, this technology results in more robust, high-quality video images.

Overall, our results in this process show that ICA has the same power to achieve efficient noise rejection in watermarking systems. With the aid of independent component analysis principles, ICA has a high potential for superiority in terms of erosion of image quality, the development of robust marking schemes, and ultimately curating high-quality yet effective watermarked images that are robust enough to be used in a wide range of applications.

### Blind source separation (BSS) results

Image watermarking is a problem that requires the use of a BSS method to address noise interference. BSS techniques provide all the necessary tools to improve the visual quality of the watermark and make it resistant to noise interference. This part presents the results of BSS algorithms applied to noise reduction, highlighting the components and analyzing their effectiveness across various platforms. Table 10 presents the results of explicit microphone noise cancellation based on BSS, including SDR, ISR, and SAR values for four BSS components (BSS1, BSS2, BSS3, BSS4).

**Table 10:** Noise reduction performance using blind source separation.

	BSS1	BSS2	BSS3	BSS4
SDR	7.941003	11.65937	12.89129	11.44235
ISR	10.037	14.12781	17.25675	12.92971
SAR	16.35647	14.33153	13.22633	18.99798

The noise-reduction results from BSS algorithms are significant across all system components. The presented results demonstrate that all chosen BSS principles significantly improve the measured metrics, including SDR, ISR, and SAR. They suggest that the BSS method is a powerful tool for dealing with various manifestations of noise and, consequently, any distortion of the watermarked source. The BSS3 component was the best pick among the factionalized groups, ranking highest across all chosen and evaluated parameters. This confirms the robustness and efficiency of blind subtractive systems in reproducing an image with significant improvement and solidity by eliminating insignificant components from noisy image data.

### Optimization results for the BSS on grayscale images

Image watermarking is a sensitive area of concern, so any noise reduction method must be precise to achieve good quality or integrity in watermarked images. As the adaptable properties of Blind Source Separation (BSS) algorithms facilitate the adoption of various enhancement technologies, performance in noise reduction improves. This part of the chapter investigates the outcomes of different optimization strategies, including Dipper-Throated Optimization (DTO), Particle Swarm Optimization (PSO), and the combined DTPSO (Dipper-Throated Particle Swarm Optimization), with the research performed on the BSS (Blind Source Separation).

The PSO-BSS employs the distributed knowledge of the flock particles to iteratively adjust the parameters of BSS algorithms, improving the specific musical features. Dimensions of the PSO-BSS demonstration are shown in Table 11, achieving better results than traditional BSS approaches. The results in SDR, ISR, and SAR are improved.

**Table 11:** The PSO-BSS Optimization Results.

	PSO-BSS1	PSO-BSS2	PSO-BSS3	PSO-BSS4
SDR	10.131	13.84937	15.08129	13.63235
ISR	12.227	16.31781	19.44675	15.11971
SAR	18.54647	16.52153	15.41633	21.18798

DTO-BSS, which stands for blind source separation, aims to modify the BSS adjustment scheme to filter out noise while preserving image features. Table 12 below presents the DTO-BSS impacts across four BSS components while continuing to improve noise reduction.

**Table 12:** The DTO-BSS Optimization Results.

SDR	DTO-BSS1	DTO-BSS2	DTO-BSS3	DTO-BSS4
	15.131	18.84937	20.08129	18.63235
ISR	17.227	21.31781	24.44675	20.11971
SAR	23.54647	21.52153	20.41633	26.18798

By combining well-established PSO with newly developed DTO, DTPSO-BSS gradually determines the optimal parameters of BSS algorithms for solving complex optimization problems. The effect of DTPSO-BSS over all four components of BSS is illustrated in Table 13, showing enhanced noise reduction performance.

**Table 13:** The DTPSO-BSS Optimization Results.

SDR	DTPSO-BSS1	DTPSO-BSS2	DTPSO-BSS3	DTPSO-BSS4
	18.8069	22.52527	23.75719	22.30825
ISR	20.9029	24.99371	28.12265	23.79561
SAR	27.22237	25.19743	24.09223	29.86388

Based on this comparison of the three optimization methods, DTPSO-BSS is the best technique for improving noise reduction in image watermarking systems. We compared all the evaluated metrics, which include SDR, ISR, and SAR, alongside DTPSO-BSS with both PSO-BSS and DTO-BSS. It was confirmed that DTPSO-BSS is a superior method for noise reduction. DTPSO-BSS stands out because this method is a blend of PSO and DTO. This amalgam allows for and is like walking along the most profitable path. The learning system determines the noise-removal threshold values in the DTPSO-BSS watermarking system; it is smoothly adjusted to noise and image features, thereby enhancing the perceptual quality and robustness of the processed images.

Table 14 shows the outcomes of the statistical analysis of different noise reduction techniques, which are Discrete Principal Component Analysis (PCA), Independent Component Analysis (ICA), Blind Source Separation (BSS), their options of optimization using Particle Swarm Optimization (PSO-BSS), Differential Transformation Optimization (DTO-BSS), and a combination of Differential Transformation and Particle Swarm Optim These methods of processing data are of great value to restricting the signal-to-noise ratio and thus good quality data is realized. In situations where signals are contaminated with noise, this becomes the only option. Statistical analysis has been conducted, including calculation of central tendency (mean, median, mode) metrics combined with dispersion (standard deviation, range, percentile) values. Furthermore, confidence intervals are provided to estimate the range of these values, along with coefficients of variation, to assess differences in the data. Skewness and kurtosis indicators provide insights into the data’s shape, which might be asymmetric or peaked. The values of these indicators can be reviewed to understand the distribution of the data.

**Table 14:** The Statistical Analysis Results.

	PCA	ICA	BSS	PSO-BSS	DTO-BSS	DTPSO-BSS
Number of values	10	10	10	10	10	10
Minimum	3.061	5.019	7.041	9.513	14.71	**18.51**
25% Percentile	3.261	5.719	7.916	10.11	15.13	**18.81**
Median	3.261	5.719	7.941	10.13	15.13	**18.81**
75% Percentile	3.39	5.769	7.941	10.13	15.13	**18.81**
Maximum	4.026	6.372	8.894	11.01	15.33	**18.91**
Range	0.9651	1.353	1.853	1.5	0.6179	**0.4**
10% Percentile	3.081	5.089	7.121	9.565	14.75	**18.54**
90% Percentile	4.001	6.327	8.799	10.92	15.31	**18.9**
Actual confidence level	97.85%	97.85%	97.85%	97.85%	97.85%	**97.85%**
Lower confidence limit	3.261	5.719	7.841	10.03	15.13	**18.81**
Upper confidence limit	3.776	5.919	7.941	10.13	15.13	**18.81**
Mean	3.369	5.734	7.936	10.15	15.11	**18.79**
Std. Deviation	0.2933	0.3256	0.4382	0.3601	0.1527	**0.1033**
Std. Error of Mean	0.09274	0.103	0.1386	0.1139	0.04829	**0.03266**
Lower 95% CI of mean	3.159	5.501	7.623	9.89	15	**18.71**
Upper 95% CI of mean	3.579	5.967	8.25	10.41	15.22	**18.86**
Coefficient of variation	8.705%	5.678%	5.521%	3.549%	1.011%	**0.5497%**
Geometric mean	3.358	5.726	7.925	10.14	15.11	**18.79**
Geometric SD factor	1.086	1.059	1.057	1.036	1.01	**1.006**
Lower 95% CI of geo. mean	3.166	5.495	7.618	9.891	15	**18.71**
Upper 95% CI of geo. mean	3.563	5.967	8.245	10.4	15.22	**18.86**
Harmonic mean	3.348	5.717	7.915	10.14	15.11	**18.79**
Lower 95% CI of harm. mean	3.171	5.487	7.613	9.892	15	**18.71**
Upper 95% CI of harm. mean	3.547	5.968	8.242	10.39	15.22	**18.86**
Quadratic mean	3.38	5.743	7.947	10.15	15.11	**18.79**
Lower 95% CI of quad. mean	3.151	5.507	7.625	9.888	15	**18.71**
Upper 95% CI of quad. mean	3.595	5.969	8.256	10.41	15.22	**18.86**
Skewness	1.724	-0.4044	0.2643	1.165	-1.995	**-2.542**
Kurtosis	2.265	3.98	4.446	5.051	6.512	**7.852**
Sum	33.69	57.34	79.36	101.5	151.1	**187.9**

**Fig. 15:**
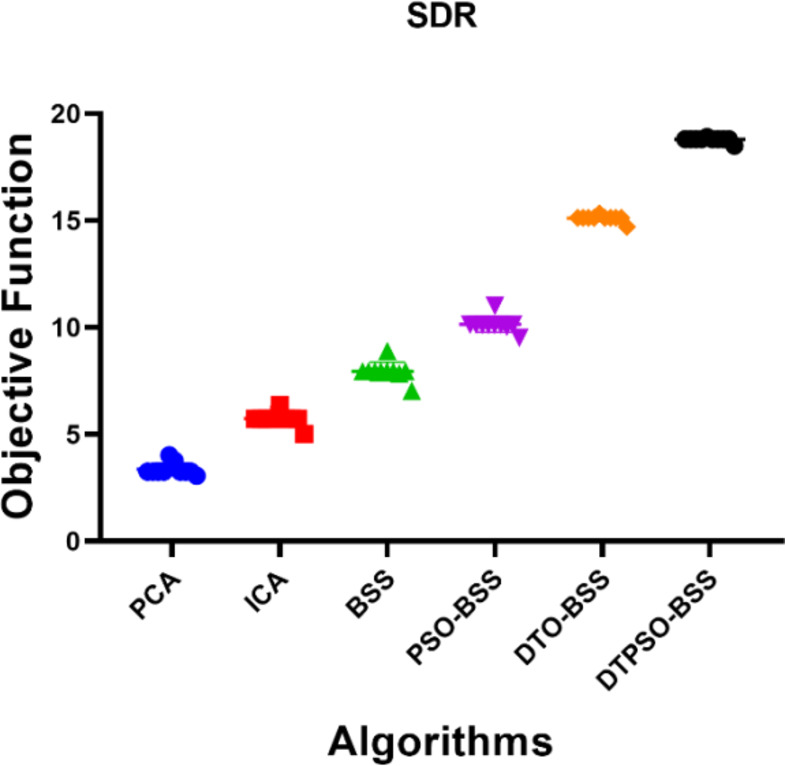
Objective function values for noise reduction methods.

Figure 15 shows the objective function values obtained for the different noise reduction methods studied in this research, displayed in a bar graph. The instruction methods to be investigated include Principal Component Analysis (PCA), Independent Component Analysis (ICA), Blind Source Separation (BSS), Particle Swarm Optimization-based BSS (PSO-BSS), Dipper-Throated Particle Swarm Optimization-based BSS (DTPSO-BSS), and Dipper-Throated Optimization-based BSS (DTO-BSS).

Table 15 presents the findings from the analysis of variation in a specific variable across different treatments or levels. ANOVA is a statistical method used to compare the means of groups with 3 or more members and determine whether there is significant variation between groups. In this table, the ANOVA analysis examines the variation in the dependent variable across different treatments using a statistical procedure.

**Table 15:** ANOVA Results for BSS Optimization Methods.

ANOVA table	SS	DF	MS	F (DFn, DFd)	*P* value
Treatment (between columns)	1696	5	339.1	F (5, 54) = 3715	*P* < 0.0001
Residual (within columns)	4.929	54	0.09128		
Total	1701	59			

**Fig. 16:**
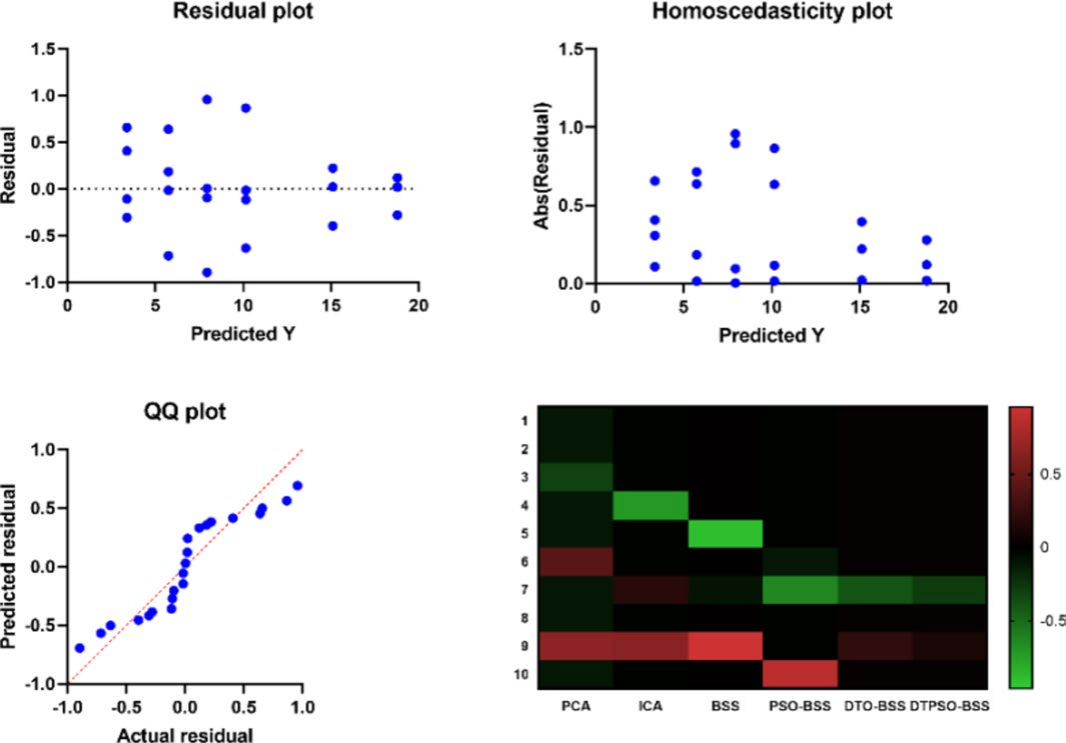
Statistical analysis plots for BSS optimization: QQ and Residual Plots.

The ANOVA analysis was followed by several visual data presentations depicting the results. For this purpose, various heatmap plots were used, as shown in Fig. 16. The QQ and residual plots were also included in these plots. The QQ plot, on the other hand, shows the positions of outliers within the distribution and confirms the exact distance between the actual and expected distributions. That line shows the `expected` distribution of data points when plotting. Residuals were plotted by hand to estimate the model’s accuracy. The presence of residuals, which are evenly distributed around zero, indicates that a model is well-calibrated and is focused on minimizing model error and maximizing fit.

The Wilcoxon Signed Rank Test is a nonparametric statistical hypothesis test that examines the null hypothesis of whether the paired samples differ at the median. In Table 16, the test is applied to compare the theoretical median (expected value) with the actual median for six different treatments: PCA, ICA, BSS (Blind Source Separation), PSO-BSS (Particle Swarm Optimization-BSS), DTO-BSS (Dipper-Throated Optimization-BSS), and DTPSO-BSS (Dipper-Throated Particle Swarm Optimization-BSS) are sub-categories of this core process.

**Table 16:** The PSO-BSS Optimization Results.

Theoretical median	PCA	ICA	BSS	PSO-BSS	DTO-BSS	DTPSO-BSS
	0	0	0	0	0	0
Actual median	3.261	5.719	7.941	10.13	15.13	**18.81**
Number of values	10	10	10	10	10	**10**
Wilcoxon Signed Rank Test						
Sum of signed ranks (W)	55	55	55	55	55	**55**
Sum of positive ranks	55	55	55	55	55	**55**
Sum of negative ranks	0	0	0	0	0	**0**
P value (two-tailed)	0.002	0.002	0.002	0.002	0.002	**0.002**
Exact or estimate?	Exact	Exact	Exact	Exact	Exact	**Exact**
P-value summary	**	**	**	**	**	**
Significant (alpha = 0.05)?	Yes	Yes	Yes	Yes	Yes	**Yes**
How significant is the discrepancy?						
Discrepancy	3.261	5.719	7.941	10.13	15.13	**18.81**

**Fig. 17:**
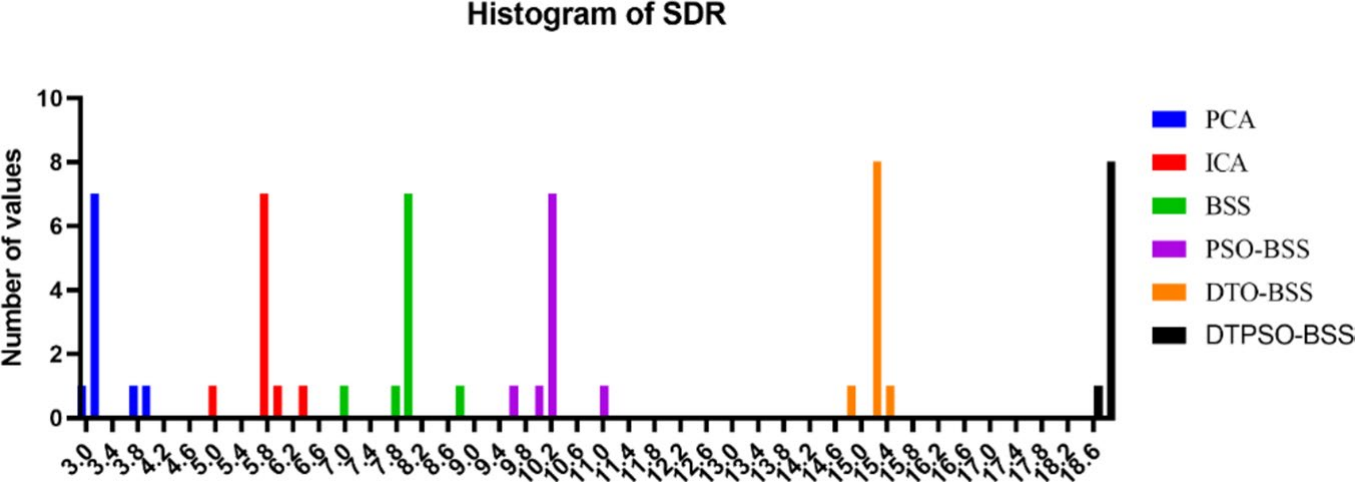
The Histogram of PSNR achieved by the proposed noise reduction techniques.

Figure 17 is a histogram of the Peak Signal-to-Noise Ratio (PSNR) obtained by the noise reduction techniques that have been proposed in this work. The methods used are classified into two branches; the first branch consists of principal component analysis(PCA), independent component analysis (ICA), and blind source separation(BSS), whereas the other branch consists of particle swarm optimization(PSO)-based BSS, dipper-throated particle swarm optimization(DTPSO)-based BSS, and dipper-throated optimization(DTO)-based BSS. The histogram represents the distribution of NPSR values obtained from these techniques. This is useful for identifying differences among the methods by analyzing the veracity of their performance. By exploring patterns in the PSNR range, readers will gain a better sense of the efficiency of these techniques.

### Optimization results for BSS on color images

**Fig. 18:**
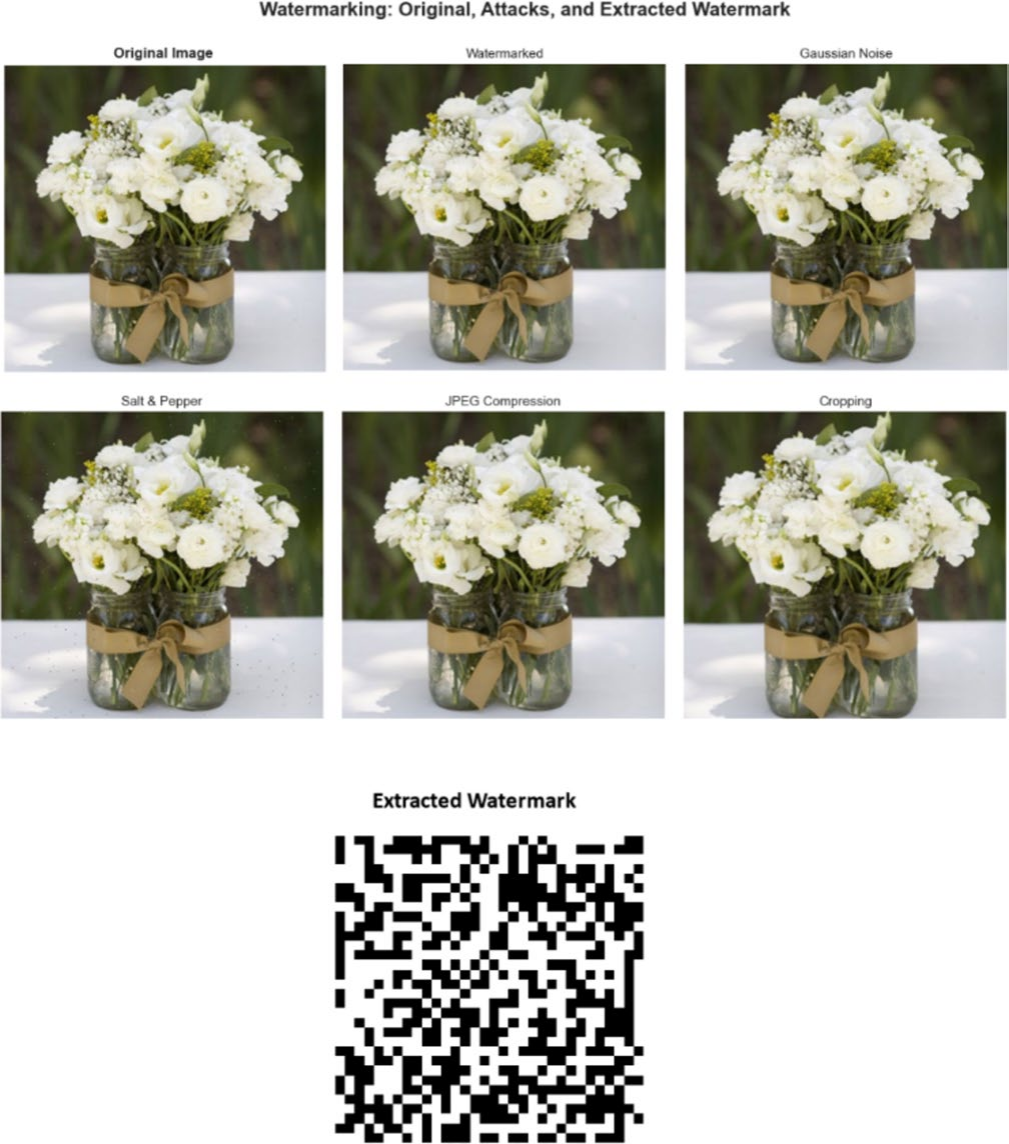
Robustness analysis of digital watermarking under multiple attacks on color image.

Figure 18 shows the successful implementation of a digital watermarking scheme designed for image protection. The sequence begins with an original image of white flowers, followed by a visually identical watermarked version, demonstrating the watermark’s imperceptibility. Subsequently, the image undergoes several common attacks – Gaussian noise, salt-and-pepper noise, JPEG compression, and cropping – each introducing varying degrees of visual distortion. Despite these manipulations, a clearly defined QR code is presented as the “Extracted Watermark”, signifying the scheme’s robustness.

Table 17 shows the model’s resilience to various attacks, focusing on metrics such as PSNR, NCC, IF, BER, and Accuracy. The model performs best under normal conditions, achieving an accuracy of 0.9766. Notably, the highest PSNR value recorded is 65.78, indicating strong image quality preservation in the absence of attacks. Across all tested attack scenarios—Gaussian Noise, Salt & Pepper, JPEG Compression, and Cropping—the model maintains a consistent level of performance. The NCC and IF metrics remain relatively stable, suggesting consistent structural similarity and feature preservation. BER remains low across all conditions, indicating minimal bit errors. While attacks do reduce performance compared to the ‘No Attack’ scenario, the model remains reasonably robust. The results highlight the model’s sensitivity to attack types, with all attacks resulting in similar performance degradation. Overall, the table provides a concise overview of the model’s vulnerability and resilience characteristics.

**Table 17:** Image quality metrics under various attack conditions on color image.

Attack	PSNR	NCC	IF	BER	Accuracy
No Attack	65.78	0.9189	0.8646	0.0234	0.9766
Gaussian Noise	59.2	0.827	0.8214	0.0429	0.9571
Salt & Pepper	59.2	0.827	0.8214	0.0429	0.9571
JPEG Compression	59.2	0.827	0.8214	0.0429	0.9571
Cropping	59.2	0.827	0.8214	0.0429	0.9571

## Conclusion and future work

This paper proposes a hybrid optimization method that blends a dipper-throated algorithm and a particle swarm algorithm to improve the performance of a watermarking system. The proposed algorithm watermarks color images using DWT and DCT, which are discrete transform tools that produce high-quality results, with PSNR of 65.78 dB, NCC of 0.9189, and accuracy of 0.9766. The low bit-error rate (BER) of 0.0234 confirms robust watermark extraction even under attack conditions. The next step in the process involves testing across 10 scenarios, demonstrating that the paper’s proposed method outperforms previous studies. Furthermore, applying the ANOVA and Wilcoxon signed-rank tests to the statistical analysis shows significant differences between our method and other approaches. The future goal of this method is to expand its functionality in the presence of noise and to develop techniques for watermarking color images in various sizes and formats.

This work thoroughly examines the latest optimization techniques that can be incorporated into image watermarking systems to reduce noise. Techniques such as AlexNet for feature extraction, PCA, ICA, and BSS can reduce noise and enhance watermarking algorithms. The experiments’ conclusions demonstrate that each method contributes to noise reduction and image quality preservation, with SDR, ISR, and SAR metrics achieving significant improvements. PCA and ICA demonstrate substantial noise reduction, while optimization techniques such as DTPSO-BSS, DTO-BSS, and PSO-BSS further enhance system performance.

In addition, the statistical analysis results show that DTPSO-BSS performs more accurately in noise reduction and maintains image quality across various noise conditions on color images. This paper explores in detail the issue of image watermarking, facilitating both research and practice in this domain. Its main contribution is the exploitation of watermarking technology for copyright protection and image authentication. Further research could include adjusting optimization algorithms, investigating the effects of watermarking across different image formats, and developing more scalable solutions for mass-image watermarking applications.

Despite the strong performance of the proposed DTPSO algorithm, it is essential to acknowledge the limitations imposed by the No Free Lunch (NFL) theorem, which states that no single optimization algorithm performs optimally across all problem domains. While DTPSO demonstrates strong results for image watermarking with DWT-DCT transforms, its effectiveness may vary when applied to different watermarking schemes or alternative application domains. Therefore, continuous algorithm refinement and problem-specific adaptations remain necessary to maintain optimal performance across diverse scenarios.

Future research directions include extending the DTPSO-optimized framework to video watermarking applications to broaden multimedia coverage. Investigating alternative deep learning architectures, such as ResNet or VGG networks, could enhance feature extraction capabilities beyond AlexNet. Additionally, developing adaptive parameter tuning mechanisms and real-time implementations for resource-constrained environments would improve practical deployment. Finally, evaluating the framework’s robustness against emerging deep learning-based attacks, including adversarial perturbations and neural network-based watermark removal techniques, remains essential for ensuring long-term security.

## Data Availability

The data used in this study are publicly available at: (https://github.com/SayedKenawy/Optimizing-Image-Watermarking-Integrity-and-Visual-Quality-via-DTPSO-and-Hybrid-Transform-Methods/tree/main?tab=readme-ov-file#readme).
